# Persistent Adult Neuroimmune Activation and Loss of Hippocampal Neurogenesis Following Adolescent Ethanol Exposure: Blockade by Exercise and the Anti-inflammatory Drug Indomethacin

**DOI:** 10.3389/fnins.2018.00200

**Published:** 2018-03-28

**Authors:** Ryan P. Vetreno, Colleen J. Lawrimore, Pamela J. Rowsey, Fulton T. Crews

**Affiliations:** ^1^Bowles Center for Alcohol Studies, School of Medicine, The University of North Carolina at Chapel Hill, Chapel Hill, NC, United States; ^2^School of Nursing, The University of North Carolina at Greensboro, Greensboro, NC, United States

**Keywords:** cytokines, alcohol, brain development, neuroprogenitors, inflammation

## Abstract

Alcohol abuse and binge drinking are common during adolescence, a developmental period characterized by heightened neuroplasticity. Animal studies reveal that adolescent ethanol exposure decreases hippocampal neurogenesis that persists into adulthood, but the mechanism remains to be fully elucidated. Using a rodent model of adolescent intermittent ethanol (AIE; 5.0 g/kg, i.g., 2-days on/2-days off from postnatal day [P]25 to P55), we tested the hypothesis that AIE-induced upregulation of neuroimmune signaling contributes to the loss of hippocampal neurogenesis in adulthood. We found that AIE caused upregulation of multiple proinflammatory Toll-like receptors (TLRs), increased expression of phosphorylated NF-κB p65 (pNF-κB p65) and the cell death marker cleaved caspase 3, and reduced markers of neurogenesis in the adult (P80) hippocampus, which is consistent with persistently increased neuroimmune signaling reducing neurogenesis. We observed a similar increase of pNF-κB p65-immunoreactive cells in the post-mortem human alcoholic hippocampus, an effect that was negatively correlated with age of drinking onset. Voluntary wheel running from P24 to P80 prevented the AIE-induced loss of neurogenesis markers (i.e., nestin and doublecortin) in the adult hippocampus that was paralleled by blockade of increased expression of the cell death marker cleaved caspase 3. Wheel running also prevented the AIE-induced increase of hippocampal pNF-κB p65 and induction of neuroimmune NF-κB target genes, including *TNF*α and *I*κ*B*α in the adult brain. Administration of the anti-inflammatory drug indomethacin during AIE prevented the loss of neurogenesis markers (i.e., nestin and doublecortin) and the concomitant increase of cleaved caspase 3, an effect that was accompanied by blockade of the increase of pNF-κB p65. Similarly, administration of the proinflammatory TLR4 activator lipopolysaccharide resulted in a loss of doublecortin that was paralleled by increased expression of cleaved caspase 3 and pNF-κB p65 in the hippocampal dentate gyrus of CON animals that mimicked the AIE-induced loss of neurogenesis. Taken together, these data suggest that exercise and anti-inflammatory drugs protect against adolescent binge ethanol-induced brain neuroimmune signaling and the loss of neurogenesis in the adult hippocampus.

## Introduction

Adolescence is a conserved neurodevelopmental period of heightened neuroplasticity that in rodents is exemplified by high levels of neurogenesis in the adolescent hippocampal dentate gyrus relative to adults (He and Crews, 2007; Curlik et al., 2014; Vetreno and Crews, 2015). Adult hippocampal neurogenesis has been implicated in cognitive and emotive processes (see e.g., Madsen et al., 2003; Revest et al., 2009). It is a dynamic process that is increased by exercise and environmental enrichment (van Praag et al., 2005) while also being decreased by pathological insults (Richardson et al., 2007) and drugs of abuse (Nixon and Crews, 2004; He et al., 2005). Preclinical rat studies reveal that hippocampal neurogenesis is more sensitive to ethanol-induced inhibition during adolescence (Crews et al., 2006), a stage of life in humans associated with high levels of binge drinking (White et al., 2006; Johnston et al., 2013). Previous adult rat studies have found that binge ethanol exposure results in a transient loss of hippocampal neurogenesis that is restored following a 30-day period of abstinence (Nixon and Crews, 2004; Broadwater et al., 2014), whereas adolescent intermittent ethanol (AIE) causes a reduction of neurogenesis in the adolescent hippocampal dentate gyrus that persists well into adulthood despite cessation of ethanol exposure (Ehlers et al., 2013a; Broadwater et al., 2014; Vetreno and Crews, 2015; Sakharkar et al., 2016). Further, AIE treatment reduces rodent hippocampal volumes in adulthood (Ehlers et al., 2013b; Vetreno et al., 2017), an effect that is also observed in human adolescents with AUDs (De Bellis et al., 2000). A similar loss of neurogenesis has been observed in a non-human primate model of adolescent binge drinking (Taffe et al., 2010). These data suggest that identifying the mechanisms underlying the persistent loss of hippocampal neurogenesis following adolescent binge drinking has important implications for understanding the development of AUD.

While the mechanism underlying the AIE-induced loss of hippocampal neurogenesis remains to be elucidated, converging evidence implicates the neuroimmune system as contributing to altered neurogenesis (Whitney et al., 2009; Mathieu et al., 2010). Systemic administration of the endotoxin lipopolysaccharide, a ligand for the proinflammatory pattern recognition receptor Toll-like receptor 4 (TLR4), reduces hippocampal neurogenesis in adult mice (Monje et al., 2003) while neurogenesis is increased in TLR4 knockout mice (Rolls et al., 2007). In hippocampal-entorhinal cortex slice culture, ethanol exposure reduces neurogenesis, and increases expression of nuclear factor kappa-light-chain-enhancer of activated B cells (NF-κB), the canonical innate immune gene transcription factor as well as several proinflammatory cytokine target genes, including *IL-1*β, *TNF*α, and *MCP-1* (Zou and Crews, 2010, 2012, 2014). Inhibition of NF-κB target genes in hippocampal-entorhinal cortex slice culture prevents ethanol-induced loss of neurogenesis (Zou and Crews, 2012). Other studies find that exposure of cultured neuroprogenitor precursor cells to proinflammatory cytokines reduces neurogenesis through increased progenitor cell death (Monje et al., 2003; Guadagno et al., 2013). Previous studies also find AIE induces adult hippocampal proinflammatory NF-κB target genes, including *MCP-1* and *TNF*α (Vetreno and Crews, 2015). These data support the hypothesis that AIE induction of neuroimmune signaling contributes to the persistent loss of adult neurogenesis.

Emerging studies find that ethanol induction of neuroimmune signaling is linked to induction of multiple neuroimmune signaling genes, including proinflammatory cytokines and their receptors, cyclooxygenase (COX), and Toll-like receptors (TLR), their endogenous agonists as well as their signaling proteins, most of which converge on NF-κB. This large number of neuroimmune signaling molecules induced by ethanol creates uncertainty in efforts to select antagonists. Aerobic exercise has repeatedly been found to recover ethanol inhibition of neurogenesis in the adult rodent hippocampus (Crews et al., 2004; Leasure and Nixon, 2010; Maynard and Leasure, 2013; Ryan and Nolan, 2016) and improve hippocampal-dependent cognitive functioning in adult humans and rodents (Griesbach et al., 2004, 2009; Hillman et al., 2008; Dery et al., 2013). We tested the hypothesis that voluntary wheel running exposure from P24 to P80 would prevent the persistent induction of neuroimmune signals and the loss of adult neurogenesis following AIE. We further tested the hypothesis that blocking neuroimmune signals using the non-steroidal anti-inflammatory drug indomethacin during AIE would prevent the loss of hippocampal neurogenesis. In the present study, we report that AIE increased adult hippocampal mRNA levels of multiple proinflammatory TLRs, increased expression of phosphorylated NF-κB p65 immunoreactive cells, and increased expression of cleaved caspase 3, a marker of cell death. In post-mortem human alcoholic hippocampal tissue, we observed a similar increase of pNF-κB p65-immunoreactive cells that was associated with an adolescent age of drinking onset. Exercise and indomethacin treatment prevented the AIE-induced loss of neurogenesis, and increased expression of neuroimmune signals and the cell death marker cleaved caspase 3. These novel findings are consistent with AIE causing lasting upregulation of neuroimmune signaling molecules that contribute to the persistent loss of hippocampal neurogenesis in adulthood. These studies also link the beneficial health effects of exercise to reduced neuroimmune signaling and increased neurogenesis.

## Methods and materials

### Animals

Male Wistar rats bred and reared at the University of North Carolina at Chapel Hill were used in this study. On the day following birth (postnatal day [P]1), litters were culled to 10 pups with six males and four females retained when possible. Pups remained with their dams in standard clear plastic tubs with shavings until group housing with same sex littermates at the time of weaning on P21. All animals were housed in a temperature- (20°C) and humidity-controlled vivarium on a 12 h/12 h light/dark cycle (light onset at 0700 h), and provided *ad libitum* access to food and water. Experimental procedures were approved by the Institutional Animal Care and Use Committee of the University of North Carolina at Chapel Hill, and conducted in accordance with National Institutes of Health regulations for the care and use of animals in research.

### Adolescent intermittent ethanol (AIE) paradigm

On P21, male Wistar rats were randomly assigned to either: (i) AIE or (ii) water control (CON) conditions. To minimize the impact of litter variables, no more than one subject from a given litter was assigned to a single experimental condition. From P25 to P55, AIE animals received a single daily intragastric (i.g.) administration of ethanol (5.0 g/kg, 20% ethanol w/v) in the AM on a 2-day on/2-day off schedule and CON subjects received comparable volumes of water. Tail blood was collected from AIE- and CON-treated subjects to assess blood ethanol concentrations (BECs) using a GM7 Analyzer (Analox; London, UK) 1 h after treatment on P38 and P54. Body weights were assessed through AIE to the conclusion of each experiment.

### Voluntary wheel running

To determine if voluntary exercise would prevent the AIE-induced loss of hippocampal neurogenesis and increased neuroimmune expression in adulthood, CON- and AIE-treated animals (*n* = 8 per group) were pair-housed on P24 in either standard cages (No exercise) or cages containing running wheels (Exercise). All subjects were observed daily for running wheel use and remained in their respective housing conditions 24 h per day. All groups were housed in pairs as social isolation can counteract the beneficial effects of exercise (Stranahan et al., 2006; Leasure and Decker, 2009). The exercise apparatus consisted of specially-designed cages with a running wheel attachment (Nalge designed for Minimitter Company, Sun River, OR). Dimensions of the fully assembled cage with the running wheel attached was 50 × 26.8 × 36.4 cm. While the running wheels did not contain a tachometer, all rats were observed running daily on the wheels. Mean BECs (±SEM) were unaffected by running wheel exposure on either P38 (AIE/No exercise: 170 ± 11 mg/dL, AIE/Exercise: 158 ± 7 mg/dL; one-way ANOVA: *p* > 0.3) or P54 (AIE/No exercise: 180 ± 12 mg/dL, AIE/Exercise: 184 ± 15 mg/dL; one-way ANOVA: *p* > 0.8). Throughout the experiment, all subjects evidenced dramatic increases in body weight across age (main effect of Age: *p* < 0.01). While we did not observe an effect of exercise or treatment on body weight during AIE, we found that no exercise CON adult (P80) animals weighed approximately 8% (~35 g) more than subjects in the other conditions. Subjects were sacrificed on P80 and brain tissue collected for assessment of neurogenesis.

### Anti-inflammatory indomethacin treatment

To determine if blockade of neuroinflammation would prevent the AIE-induced loss of hippocampal neurogenesis, indomethacin (4.0 mg/kg, i.p., Sigma-Aldrich, St. Louis, MO) was administered to CON- and AIE-treated animals (*n* = 8 per group) 30 min before treatment on a 2-day on/2-day off schedule. Indomethacin was suspended in 0.1 mL dimethyl sulfoxide and brought to a concentration of 0.8 mg/mL with 0.9% saline (pH: 7.4). Since our laboratory previously found that AIE reduced neurogenesis in the adolescent hippocampus that persisted into adulthood (Vetreno and Crews, 2015), subjects were sacrificed 24 h following the conclusion of AIE (P56) and brain tissue collected for assessment of neurogenesis.

### Lipopolysaccharide (LPS) treatment protocol

To determine if administration of the proinflammatory TLR4 activator LPS would diminish hippocampal neurogenesis, and increase expression of cleaved caspase 3 and pNF-κB p65, a separate group of CON- and AIE-treated animals (*n* = 8 per group) received a single injection of LPS (1.0 mg/kg in sterile 0.9% saline, i.p., E. Coli, serotype 0111:B4; Sigma-Aldrich) or a comparable volume of saline on P70. Subjects were sacrificed 10 days later on P80 and brain tissue collected for assessment of neurogenesis.

### Perfusion and tissue collection

At the conclusion of each experiment, subjects were anesthetized with a euthanasia dose of sodium pentobarbital (100 mg/kg, i.p.) and transcardially perfused with 0.1 M phosphate-buffered saline (PBS, pH 7.4). In the exercise experiments, brain, liver, and spleen samples were collected, rapidly frozen in liquid nitrogen, and stored at −80°C for protein and mRNA extraction. For the immunohistochemistry experiments, subjects were transcardially perfused with 0.1 M PBS (pH 7.4) followed by 4.0% paraformaldehyde in PBS. Brains were excised and post-fixed in 4.0% paraformaldehyde for 24 h at 4°C followed by 4 days of fixation in 30% sucrose solution. Coronal sections were cut (40 μm) on a sliding microtome (MICROM HM450; ThermoScientific, Austin, TX), and sections were sequentially collected into well plates and stored at −20°C in a cryoprotectant solution (30% glycol/30% ethylene glycol in PBS).

### Immunohistochemistry in animal studies

Free-floating hippocampal sections (every 12th section) were washed in 0.1 M PBS, incubated in 0.3% H_2_O_2_ to inhibit endogenous peroxidases, and blocked with the appropriate normal serum (MP Biomedicals, Solon, OH). Sections were incubated in a primary antibody solution containing blocking solution, and either goat anti-doublecortin (Santa Cruz Biotechnology, Santa Cruz, CA; Cat. #sc-8066), rabbit anti-Ki-67 (Abcam, Cambridge, MA; Cat. #ab66155), rabbit anti-cleaved caspase 3 (Cell Signaling Technology, Danvers, MA; Cat. #9661), rabbit anti-phosphorylated NF-κB p65 Ser 276 (pNF-κB p65; Santa Cruz Biotechnology; Cat. #sc-101749), or mouse anti-nestin (Millipore, Temecula, CA; Cat. #MAB5326) for 24 hr at 4°C. Sections were then washed with PBS, incubated for 1 h in the appropriate biotinylated secondary antibody (Vector Laboratories, Burlingame, CA), and incubated for 1 h in avidin-biotin complex solution (Vectastain ABC Kit; Vector Laboratories). The chromogen, nickel-enhanced diaminobenzidine (Sigma-Aldrich, St. Louis, MO), was used to visualize immunoreactivity. Tissue was mounted onto slides, dehydrated, and cover slipped. Negative control for non-specific binding was conducted on separate sections employing the abovementioned procedures omitting the primary antibody.

### Post-mortem human hippocampal tissue samples

Post-mortem human paraffin sections of the hippocampus from moderate drinking control and alcoholic subjects were obtained from the New South Wales Tissue Resource Centre (NSWTRC [ethnics committee approval number: X11-0107]) at the University of Sydney (supported by the National Health and Medical Research Council of Australia-Schizophrenia Research Institute and the National Institute of Alcohol Abuse and Alcoholism [NIH (NIAAA) R24AA012725]). Subject information was collected through personal interviews, next-of-kin interviews, and medical records, and is presented in Table 1. Since establishing an accurate age of drinking onset is critical, trained clinical nurses, and psychologists from the NSWTRC performed extensive interviews with the human volunteers and their families. Age of drinking onset was derived from personal interviews with the volunteers as well as medical records and next-of-kin interviews. In cases where the age of drinking onset was unclear, an age of 25 was recorded (Sheedy et al., 2008). Alcoholic subjects reported an average age of drinking onset of 17 (±1), which was compared to age-matched moderate drinking controls whose average age of drinking onset was 25 (±1). It is noteworthy that 100% of alcoholics were able to recall their age of drinking onset whereas only 10% of moderate drinking controls were able to provide accurate information. Only individuals with alcohol dependence uncomplicated by liver cirrhosis and/or nutritional deficiencies were included in the present study. All psychiatric and alcohol use disorder diagnoses were confirmed using the Diagnostic Instrument for Brain Studies that complies with the Diagnostic and Statistical Manual of Mental Disorders (Dedova et al., 2009).

**Table 1:** Case characteristics of male human subjects used for IHC analysis in the post-mortem hippocampus.

**Subject ID**	**Group**	**PMI**	**Clinical cause of death**	**Age of drinking onset**
635	Control	40	Haemopericardium	25
301	Control	16	Dilated cardiomyopathy	25
395	Control	28	Ischemic heart disease	25
629	Control	46	Ischemic heart disease	25
498	Control	43	Undetermined (Consistent with idiopathic cardiac arrhythmia)	20
335	Control	50	Ischemic heart disease	25
453	Control	29	Acute myocardial infarction	25
329	Control	24	Ischemic heart disease	25
670	Alcoholic	16	Coronary artery thrombosis	14
287	Alcoholic	17	Ischemic heart disease	18
210	Alcoholic	17	Gastrointestinal hemorrhage	17
643	Alcoholic	59	Myocarditis	16
679	Alcoholic	23.5	Atherosclerotic cardiovascular disease	17
286	Alcoholic	43.5	Carbon monoxide intoxication/Alcohol intoxication	16
591	Alcoholic	18.5	Drowning	15
654	Alcoholic	44	Ischemic heart disease	16

*PMI, post-mortem interval. Mean age of drinking onset for moderate drinking controls was 25 (±1) and for alcoholics was 17 (±1). Alcoholic individuals tended to demonstrate an earlier age of drinking onset in comparison to moderate drinking controls*.

### Human immunohistochemistry

Paraffin-embedded human post-mortem hippocampal sections were deparaffinized, washed in PBS, and antigen retrieval performed by incubation in Citra solution (BioGenex, San Ramon, CA) for 1 h at 70°C. Following incubation in normal goat serum (MP Biomedicals) blocking solution, slides were incubated in a primary antibody solution containing rabbit anti-pNF-κB p65 Ser 276 (Santa Cruz Biotechnology) for 24 h at 4°C. Slides were incubated for 1 h in biotinylated secondary antibody (1:200; Vector Laboratories) and then for 1 h in avidin–biotin complex solution (Vector Laboratories). Nickel-enhanced DAB (Sigma-Aldrich) was used to visualize immunoreactivity. Slides were dehydrated and cover slipped. Negative control for non-specific binding was conducted employing the above-mentioned procedures with omission of the antibody.

### Microscopic quantification and image analysis

Across experiments, BioQuant Nova Advanced Image Analysis software (R&M Biometric, Nashville, TN) was used for image capture and quantification of immunohistochemistry. Representative images were captured using an Olympus BX50 microscope and Sony DXC-390 video camera linked to a computer. For each measure, the microscope, camera, and software were background corrected, and normalized to preset light levels to ensure fidelity of data acquisition.

Immunohistological assessment was performed in the rat dorsal hippocampal dentate gyrus according to the atlas of Paxinos and Watson (1998). Pixel density was used to assess doublecortin immunoreactivity (+IR) as it is densely distributed throughout the subgranular zone of the hippocampal dentate gyrus making identification of individual neurons difficult. Pixel density was rigorously thresholded to normalize pixel intensity (Vetreno and Crews, 2015). The threshold for pixel density was determined from control subjects by calculating the average of the darkest and lightest values, and sections were imaged under identical conditions to avoid non-systematic variation (Beynon and Walker, 2012). The outlined regions of interest were determined and pixel density calculated by dividing the pixel count by the overall area (mm^2^). A modified stereological quantification method was used to quantify Ki-67, cleaved caspase 3, pNF-κB p65, and nestin immunopositive cells in the rat hippocampal dentate gyrus, and pNF-κB p65 immunopositive cells in the human hippocampal dentate gyrus. We previously reported that a comparison of unbiased stereological methodology with our modified stereological approach yielded nearly identical values relative to control subjects (Crews et al., 2004). The outlined regions of interest were determined and data expressed as cells/mm^2^. Brain sections from all groups in each experiment are stained simultaneously to control for differences in antibody lots, DAB staining, time after sectioning, and other factors that can affect staining intensity. Thus, absolute immunohistochemical control values (cell counts and pixel density) sometimes vary between experiments, however, the relationships across experimental groups remain consistent.

### Immunofluorescence and colocalization analysis

To assess pNF-κB p65 colocalization, free-floating hippocampal sections were processed similar to previously reported methods (Vetreno et al., 2013; Vetreno and Crews, 2015). Briefly, sections collected from subjects sacrificed on P56 (24 h post-AIE) were washed in 0.1 M TBS, antigen retrieval performed by incubation in Citra solution (BioGenex]) for 1 h at 70°C, and blocked with normal horse serum (MP Biomedicals). Sections were incubated for 48 h at 4°C in a primary antibody cocktail of rabbit anti-pNF-κB p65 (Santa Cruz Biotechnology) with an antibody against immature neurons (goat anti-doublecortin [Santa Cruz Biotechnology]), microglia (goat anti-Iba-1 [Abcam; Cat. #ab5076]), or astrocytes (goat anti-GFAP [Abcam; Cat. #ab53554]). Sections were washed in TBS and incubated for 2 h at room temperature in the secondary antibody cocktail (Alexa Fluor 594 and Alexa Fluor 488; Invitrogen, Carlsbad, CA). Tissue was mounted onto slides and cover slipped using Prolong Gold Anti-Fade mounting media (Life Technologies, Grand Island, NY). Immunofluorescent images were obtained using a DS-RiZ scope (Nikon Inc., Melville, NY) and colocalization quantified using NIS Elements AR46 (Nikon Inc.).

### RNA extraction and reverse transcription polymerase chain reaction (RTPCR)

For assessment of TLR mRNA expression, hippocampal samples were collected from adult (P80) CON- and AIE-treated animals according to the atlas of Paxinos and Watson (1998). To determine the effect of exercise on AIE-induced neuroimmune expression, hemi-sectioned whole brain tissue (referred to as whole brain hence forth) and spleen samples were collected from CON- and AIE-treated animals on P80. Across experiments, total mRNA was extracted from each sample by homogenization in TRI reagent (Sigma-Aldrich) following the single-step method of RNA isolation (Chomczynski and Sacchi, 2006). Total mRNA was reverse transcribed as previously described (Vetreno and Crews, 2012; Vetreno et al., 2013). SYBER green PCR Master Mix (Life Technologies, Carlsbad, CA) was used for the RT-PCR. The real-time PCR was run with an initial activation for 10 min at 95°C, followed by 40 cycles of denaturation (95°C, 15 s), annealing/extension (57–58°C, 1 min), and finally a melt curve. The primer sequences are presented in Table 2. Differences in primer expression between groups are expressed as cycle time (Ct) values normalized with β-actin, and relative differences between groups calculated and expressed as the percent difference relative to CONs.

**Table 2:** List of primers for rat qPCR.

**Primer**	**Forward**	**Reverse**
*TLR1*	5′-TAC CCT GAA CAA CGT GGA CA-3′	5′-ATC GAC AAA GCC CTC AGA GA-3′
*TLR2*	5′-GGA GAC TCT GGA AGC AGG TC-3′	5′-CGC CTA AGA GCA GGA TCA AC-3′
*TLR3*	5′-GCA ACA ACA ACA TAG CCA AC-3′	5′-CCT TCA GGA AAT TAA CGG GAC-3′
*TLR4*	5′-CCA GAG CCG TTG GTG TAT CT-3′	5′-CCA GAG CCG TTG GTG TAT CT-3′
*TLR5*	5′-CAG TTG CGA ACC ATA AGG ACG-3′	5′-GAG GTC ACC GAG ACA AAG CAC-3′
*TLR6*	5′-GTC TCC CCA CTT CAT CCA GA-3′	5′-CCC ACG TTT ACC CTT CTC AA-3′
*TLR7*	5′-AGC TCT GTT CTC CTC CAC CA-3′	5′-CAT GGG TGT TTG TGC TAT CG-3′
*TLR8*	5′-CTT CCA AAC TTG GCA ACC AT-3′	5′-GAA GAC GAT TTC GCC AAG AG-3′
*TLR9*	5′-TCA ACA AGA ACA CGC TCA GG-3′	5′-GAG AGC TGG GGT GAG ACT T-3′
*HMGB1*	5′-ATG GGC AAA GGA GAT CCT A-3′	5′-ATT CAT CAT CAT CAT CTT CT-3′
*TNFα*	5′-ATG TGG AAC TGG CAG AAG AG-3′	5′-ACG AGC AGG AAT GAG AAG AAG-3′
*MCP1*	5′-CTG GGC CTG TTG TTC ACA GTT GC-3′	5′-CTA CAG AAG TGC TTG AGG TGG TTG-3′
*IL-1β*	5′-GAA ACA GCA ATG GTC GGG AC-3′	5′-AAG ACA CGG GTT CCA TGG TG-3′
*IL-4*	5′-TGA ATG AGT CCA CGC TCA CA-3′	5′-GTT AGG ACA TGG AAG TGC AGG-3′
*IL-10*	5′-GAA CCA CCC GGC ATC TAC TG-3′	5′-TGG AGA GAG GTA CAA ACG AGG-3′
*TGFβ*	5′-GCT GAA CCA AGG AGA CGG AA-3′	5′-CCA CGT AGT AGA CGA TGG GC-3′
*IκBα*	5′-CGT GGA GCA CTT GGT GAC TT-3′	5′-AAG CTG GTA GGG GGA GTA GC-3′
*β-actin*	5′-CTA CAA TGA GCT GCG TGT GGC-3′	5′-CAG GTC CAG ACG CAG GAT GGC-3′

### Enzyme-linked immunosorbent assay (ELISA) for HMGB1 and IL-1β

Frozen liver samples were homogenized in cold lysis buffer (100 mg tissue/mL; 20 mM Tris, 0.25 M sucrose, 2.0 mM EDTA, 10 mM EGTA, 1.0% Triton X-100) containing Complete Mini protease inhibitor cocktail (one tablet/10 mL; Roche Diagnostics, Indianapolis, IN). Liver homogenates were centrifuged at 100,000 RPM at 4°C for 40 min, supernatant collected, and protein levels determined using the BCA protein assay kit (Pierce, Milwaukee, WI). Levels of HMGB1 (IBL, Hamburg, Germany) and IL-1β (R&D Systems, Minneapolis, MN) were assessed according to the manufacturer's protocol.

Tail blood was collected into heparin-coated tubes from rats on P54. Plasma was isolated by centrifugation of blood samples twice at 16,000 × g for 20 min at 4°C. Levels of HMGB1 were assessed following manufacturer's protocol.

### Statistical analysis

Statistical analysis was performed using SPSS (Chicago, IL). One-way analysis of variance (ANOVA) was used to assess BECs. Separate 2 × 2 ANOVAs were used to assess body weight, immunohistochemistry, RT-PCR, and ELISA data. *Post-hoc* analyses were performed using Tukey's HSD where appropriate. All values are reported as mean ± SEM, and significance was defined as *p* ≤ 0.05.

## Results

### Adolescent intermittent ethanol treatment causes long-term upregulation of toll-like receptors (TLR) in the adult hippocampus

**Figure 1:**
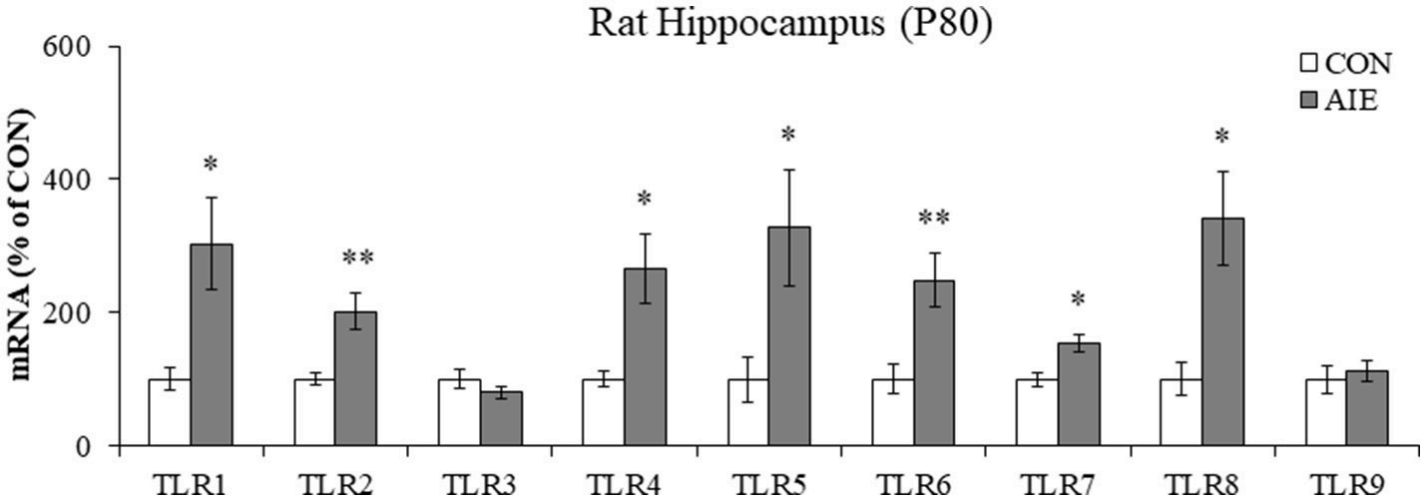
Adolescent intermittent ethanol (AIE) treatment upregulated expression of Toll-like receptor (TLR) genes in the adult hippocampus. Quantitative PCR assessment of TLR mRNA in adult (P80) hippocampal tissue samples revealed an approximate 3-fold increase of *TLR1, TLR5*, and *TLR8*, an approximate 2.5-fold increase of *TLR4* and *TLR6*, an approximate 2-fold increase of *TLR2*, and an approximate 50% increase of *TLR7*, relative to CONs. We did not observe an effect of AIE on mRNA levels of *TLR3* or *TLR9*. qPCR analyses were run in triplicate. Data are presented as mean ± SEM. ^*^p < 0.05, ^**^p < 0.01 relative to CON.

Emerging studies have found that TLRs are linked to ethanol induction of neuroimmune signaling (Crews and Vetreno, 2016; Crews et al., 2017). To determine if AIE causes long-term increases in TLR expression, we measured TLR mRNA in the adult hippocampus (P80) 25 days after the last binge ethanol exposure. We found that AIE treatment significantly increased mRNA expression of *TLR1* [303% [±69%]; one-way ANOVA: *F*_(1, 10)_ = 8.3, *p* < 0.05], *TLR2* [202% [±27%]; one-way ANOVA: *F*_(1, 10)_ = 12.2, *p* < 0.01), *TLR4* (266% [±53%]; one-way ANOVA: *F*_(1, 10)_ = 9.4, *p* < 0.05], *TLR5* [328% [±87%]; one-way ANOVA: *F*_(1, 10)_ = 6.0, *p* < 0.05), *TLR6* (249% [±41%]; one-way ANOVA: *F*_(1, 10)_ = 10.2, *p* < 0.01], *TLR7* [154% [±14%]; one-way ANOVA: *F*_(1, 10)_ = 9.8, *p* < 0.05], and *TLR8* [341% [±71%]; one-way ANOVA: *F*_(1, 10)_ = 8.6, *p* < 0.05], relative to CONs (see Figure 1). Levels of *TLR3* and *TLR9* were unchanged in the adult hippocampus following AIE exposure (both *p*'s > 0.2). These data reveal that AIE treatment leads to long-term upregulation of proinflammatory TLR expression in the adult hippocampus.

### Phosphorylated NF-κB p65 immunoreactivity is increased in the hippocampal dentate gyrus of AIE-treated adult rats and post-mortem human alcoholics

**Figure 2:**
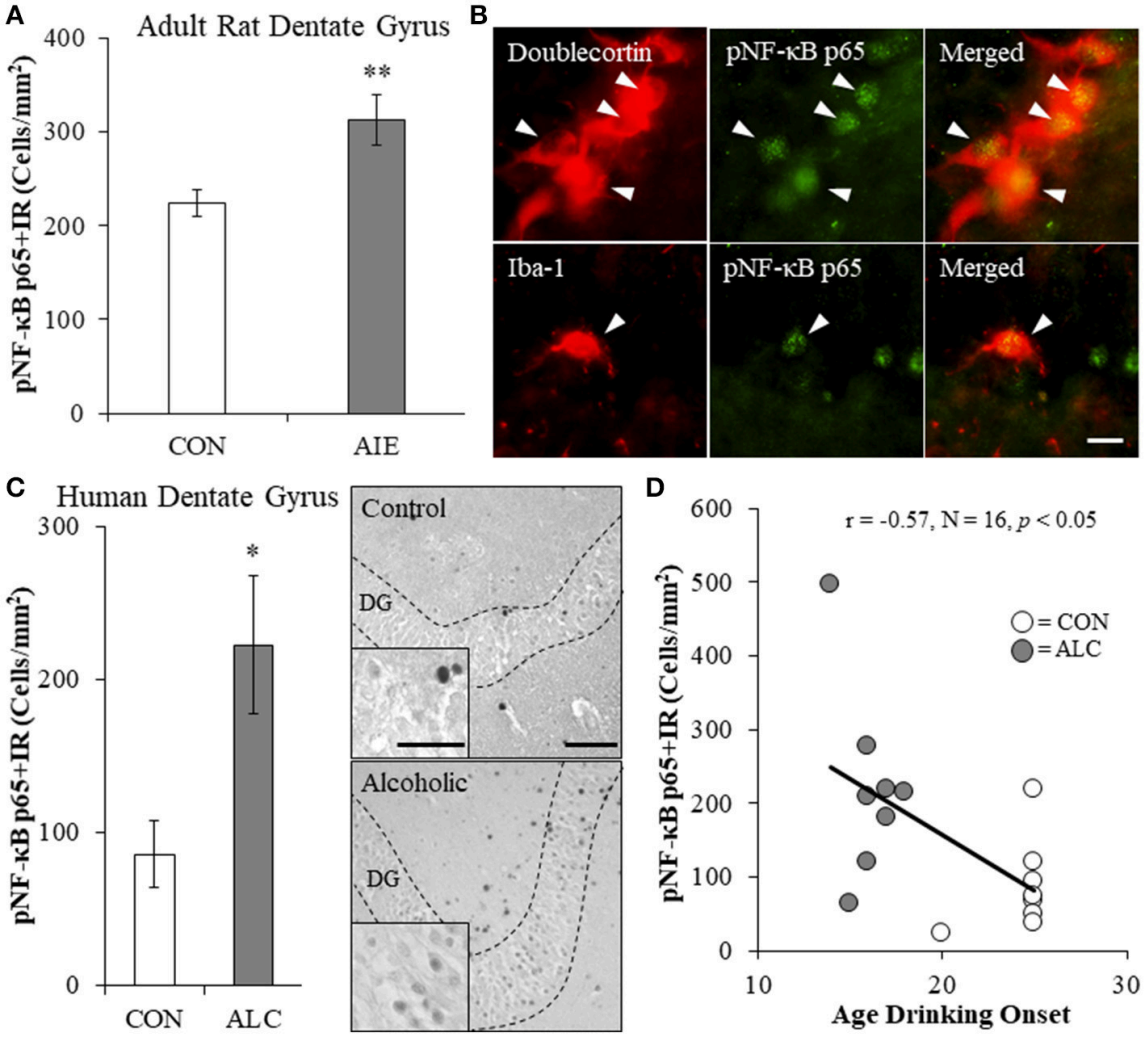
Increased expression of the proinflammatory transcription factor nuclear factor kappa-light-chain-enhancer of activated B cells p65 (phosphorylated [pNF-κB p65]) in the hippocampal dentate gyrus of adolescent intermittent ethanol (AIE)-treated adult rats and post-mortem human alcoholics. **(A)** Modified unbiased stereological quantification of pNF-κB p65+IR cells revealed a 40% (±12%) increase in the adult (P80) hippocampal dentate gyrus of AIE-treated animals, relative to CONs. **(B)** High magnification photomicrographs of pNF-κB p65 (green) colocalization with the immature neuron marker doublecortin and the microglial marker Iba-1 (red) in the hippocampal granule cell layer of an AIE-treated rat. White arrowheads indicate pNF-κB p65+IR cells that colocalized with a cellular marker. Scale bar = 10 μm. **(C)** pNF-κB p65+IR was increased in the post-mortem human alcoholic (ALC) hippocampal dentate gyrus (*n* = 8), relative to moderate drinking controls (CON; *n* = 8). Representative photomicrographs of pNF-κB p65+IR in a moderate drinking control and alcoholic subject. Dashed lines indicate outline of the granule cell layer of the hippocampal dentate gyrus. Scale bar = 25 μm (Insert: 10 μm). **(D)** Across subjects, age of drinking onset was negatively correlated with expression of pNF-κB p65+IR cells in the hippocampal dentate gyrus (*r* = −0.57, *N* = 16, *p* < 0.05). Moderate alcohol drinking controls tended to report a later age of drinking onset (25 ± 1 years of age) relative to individuals that met criteria for alcoholism (17 ± 1 years of age). ^*^p < 0.05, ^**^p < 0.01, relative to CONs.

Since AIE led to the long-term induction of multiple TLRs and NF-κB is a common transcription factor target of TLR activation, we next assessed expression of pNF-κB p65+IR in the hippocampal dentate gyrus of adult rats (P80) following AIE treatment. Phosphorylated NF-κB p65+IR was characterized by darkly stained cell nuclei throughout the dentate gyrus. Analysis of pNF-κB p65+IR revealed a significant 40% (±12%) increase in the adult AIE-treated animals, relative to CONs [one-way ANOVA: *F*_(1, 14)_ = 8.7, *p* < 0.01; see Figure 2A]. Colocalization analysis revealed a high degree of pNF-κB p65 co-expression with doublecortin (CON: 54% [±3%]; AIE: 55% [±4%]), which did not differ as a function of treatment (*p* > 0.8). Interestingly, we found that 26% (±3%) of Iba-1+IR microglia in the AIE dentate gyrus colocalized with pNF-κB p65 whereas only 8% (±2%) colocalized in the CONs [one-way ANOVA: *F*_(1, 8)_ = 28.3, *p* < 0.01; see Figure 2B]. In contrast, we observed minimal GFAP colocalization with pNF-κB p65 in either treatment group. These data suggest that increased pNF-κB p65+IR in the adult hippocampus following AIE is associated with increased expression in microglia.

We next determined whether pNF-kB p65+IR is increased in the post-mortem human alcoholic hippocampal dentate gyrus. We found a 160% (±53%) increase of pNF-κB p65+IR in the alcoholic dentate gyrus, relative to moderate drinking controls [one-way ANOVA: *F*_(1, 14)_ = 7.4, *p* < 0.05; see Figure 2C]. Since an earlier age of drinking onset is associated with an increased risk of lifetime alcohol dependence (Gruber et al., 1996; Grant and Dawson, 1998), we correlated age of drinking onset with pNF-κB p65+IR. Drinking age was negatively correlated with pNF-κB p65+IR (*r* = −0.57, *N* = 16, *p* < 0.05) indicating that an earlier age of drinking onset is associated with increased expression of pNF-κB p65+IR in the hippocampus (see Figure 2D. Together, these data suggest that adolescent exposure to alcohol results in a persistent increase in dentate gyrus pNF-κB p65+IR in adult AIE-treated rats and in human alcoholics.

### Voluntary exercise prevents the AIE-induced loss of progenitor markers and neurogenesis in the adult hippocampal dentate gyrus

**Figure 3:**
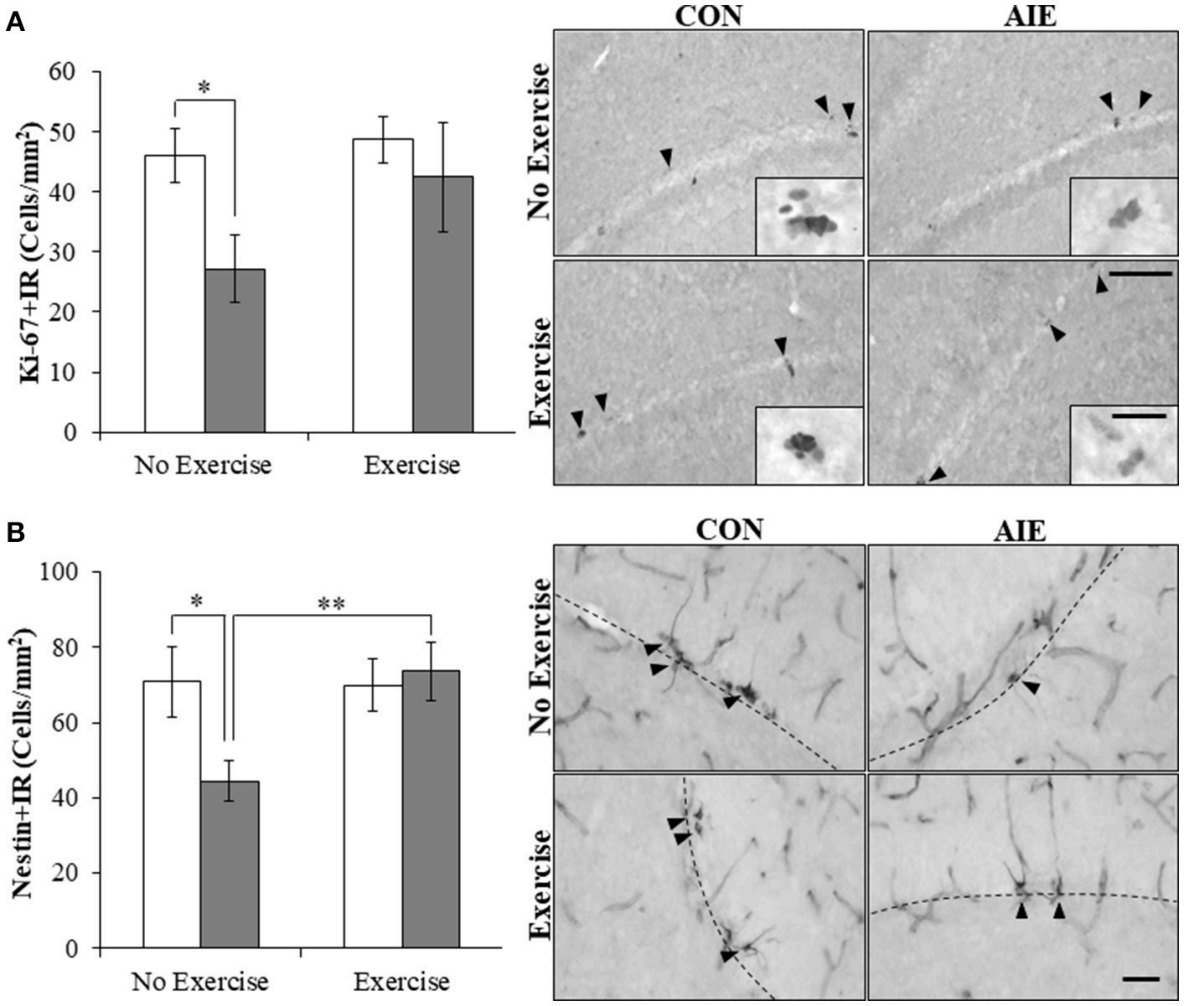
Voluntary wheel running prevented the adolescent intermittent ethanol (AIE)-induced loss of neuroprogenitor marker expression in the adult hippocampal dentate gyrus. **(A)** Modified unbiased stereological assessment revealed a 41% (±12%) reduction of Ki-67+IR cells in the adult (P80) hippocampal dentate gyrus of AIE-treated animals, relative to CONs. Running wheel exposure from P24 to P80 blunted the AIE-induced loss of Ki-67+IR cells, relative to the AIE animals. Representative photomicrographs of Ki-67+IR cells in the adult hippocampal dentate gyrus from CON- and AIE-treated animals across exercise conditions. Scale bar = 100 μm (Insert: 10 μm). **(B)** Modified unbiased stereological assessment revealed a 37% (±8%) reduction of nestin+IR cells in the adult hippocampal dentate gyrus of AIE-treated animals, relative to CONs. Subjects in the AIE treatment group that were exposed to running wheels from P24 to P80 did not evidence the observed loss of nestin+IR cells, relative to the AIE animals. Representative photomicrographs of nestin+IR cells in the adult hippocampal dentate gyrus from CON- and AIE-treated animals across exercise conditions. Scale bar = 50 μm. Dashed lines indicate outline of the granule cell layer of the hippocampal dentate gyrus. Data are presented as mean ± SEM. ^*^p < 0.05, ^**^p < 0.01.

Multiple studies have found that ethanol inhibition of hippocampal neurogenesis is related to NF-κB activation, reduced progenitor proliferation, and increased cell death (Crews and Nixon, 2009). To determine if AIE combined with voluntary exercise would prevent the AIE-induced loss of neurogenesis, we exposed CON- and AIE-treated animals to running wheels. Expression of the progenitor proliferation marker Ki-67 (Scholzen and Gerdes, 2000) was characterized by darkly stained clusters of cell bodies within the subgranular zone of the hippocampus. Analysis with a 2 × 2 ANOVA (Treatment [CON vs. AIE] × Exercise [No exercise vs. Exercise]) revealed that AIE treatment significantly reduced Ki-67+IR cells in the adult hippocampus (main effect of Treatment: *F*_(1, 28_) = 4.2, *p* ≤ 0.05). While a significant interaction of Treatment × Exercise was not observed, follow-up analysis revealed a 41% (±12%) reduction of Ki-67+IR cells in the AIE-treated animals, relative to CONs [one-way ANOVA: *F*_(1, 14)_ = 6.8, *p* < 0.05; see Figure 3A]. Further, we found that wheel running blunted, albeit insignificantly, the AIE-induced reduction of Ki-67+IR (*p* = 0.17). While assessment the progenitor marker nestin (Palmer et al., 2000) with a 2 × 2 ANOVA did not yield a significant interaction, follow-up analyses revealed that nestin expression was reduced by 37% (±8%) in the AIE-treated animals, relative to the CONs [one-way ANOVA: *F*_(1, 13)_ = 5.4, *p* < 0.05; see Figure 3B]. Interestingly, voluntary wheel running prevented the AIE-induced loss of nestin+IR cells [one-way ANOVA: *F*_(1, 13)_ = 9.3, *p* < 0.01]. Thus, voluntary exercise blunted the AIE-induced loss of the proliferation marker Ki-67 and prevented the reduction of nestin+IR cells in the adult hippocampal dentate gyrus.

**Figure 4:**
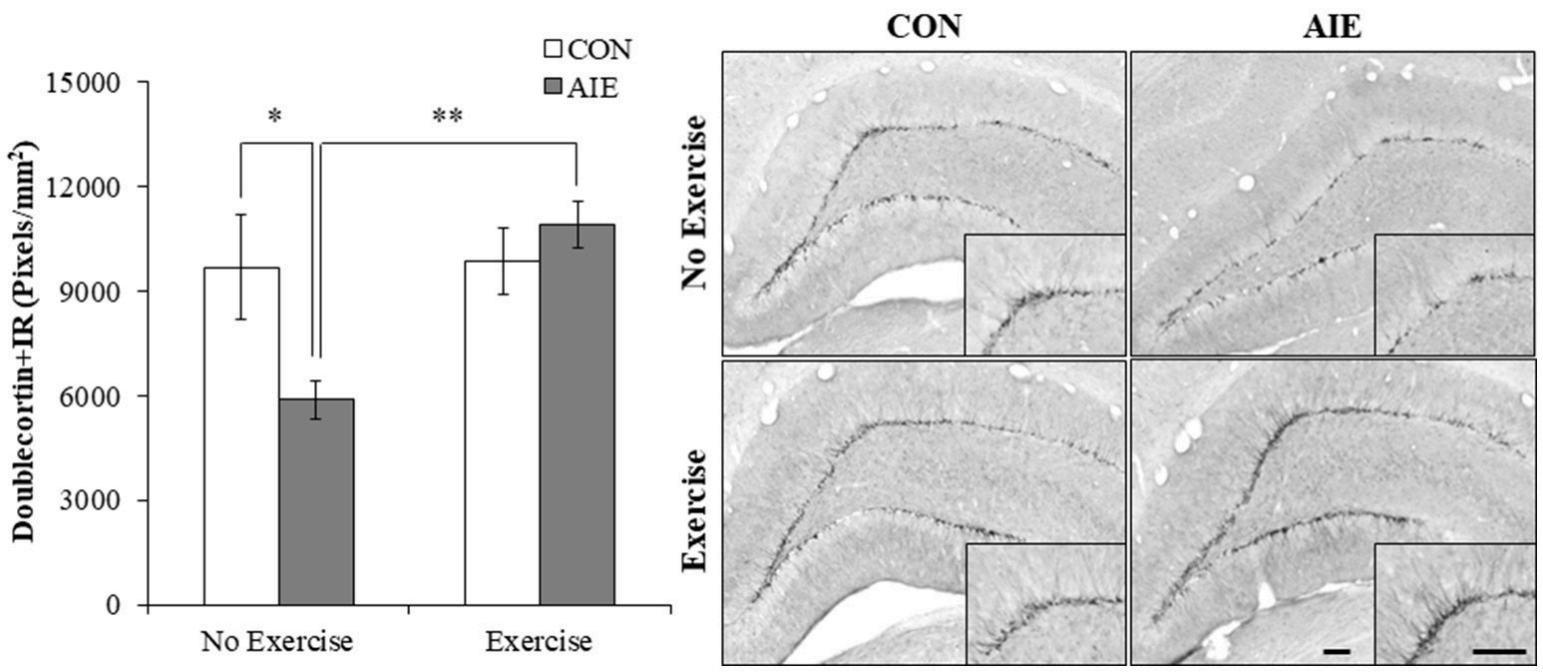
Voluntary exercise prevented the adolescent intermittent ethanol (AIE)-induced loss of the immature neuron marker doublecortin in the adult hippocampal dentate gyrus. Pixel density quantification revealed a 36% (±6%) reduction of doublecortin+IR in the adult (P80) hippocampal dentate gyrus of AIE-treated animals, relative to CONs. Subjects in the AIE treatment group that were exposed to running wheels from P24 to P80 did not evidence the observed loss of doublecortin+IR, relative to standard housed AIE animals. Representative photomicrographs of doublecortin+IR in the adult hippocampal dentate gyrus from CON- and AIE-treated animals across exercise conditions. Scale bar = 100 μm (Insert: 100 μm). Data are presented as mean ± SEM. ^*^p < 0.05, ^**^p < 0.01.

We next assessed expression of doublecortin, a microtubule protein expressed by immature dentate gyrus neurons that provides an index of overall neurogenesis (Brown et al., 2003; Couillard-Despres et al., 2005). Doublecortin immunoreactivity was characterized by darkly stained cell bodies in the subgranular zone with processes that innervated the granular cell layer of the dentate gyrus. A 2 × 2 ANOVA (Treatment × Exercise) revealed that wheel running significantly increased doublecortin+IR in the hippocampus [main effect of Exercise: *F*_(1, 28)_ = 7.0, *p* < 0.05]. *Post-hoc* analysis of the significant Treatment × Exercise interaction [*F*_(1, 28)_ = 6.1, *p* < 0.05] revealed that doublecortin+IR was reduced by 36% (±6%) in the AIE-treated animals (Tukey's HSD: *p* ≤ 0.05), relative to CONs. While wheel running alone did not affect doublecortin+IR, exercise combined with AIE prevented the loss of doublecortin in the adult hippocampus (Tukey's HSD: *p* < 0.01; see Figure 4). Together, these data reveal that wheel running prevents the AIE-induced reduction of dentate gyrus progenitor markers and loss of neurogenesis in the adult hippocampus.

### Wheel running prevents the AIE-induced increase of cleaved caspase 3 in the adult hippocampal dentate gyrus

**Figure 5:**
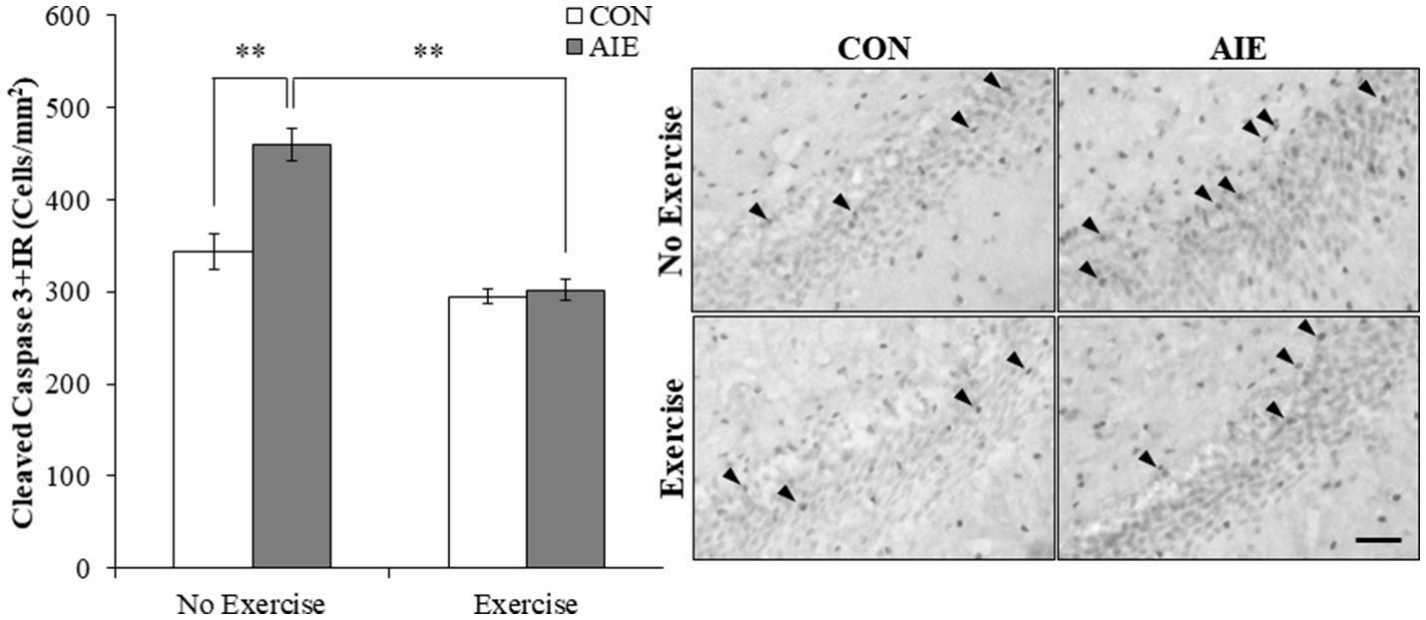
Running wheel exposure prevented the adolescent intermittent ethanol (AIE)-induced increase of the cell death marker cleaved caspase 3 in the adult hippocampal dentate gyrus. Modified unbiased stereological quantification revealed a 34% (±5%) increase of cleaved caspase 3+IR cells in the adult (P80) hippocampal dentate gyrus of AIE-treated animals, relative to CONs. Voluntary wheel running from P24 to P80 prevented the AIE-induced increase of cleaved caspase 3+IR cells, relative to AIE animals. Representative photomicrographs of cleaved caspase 3+IR cells in the adult hippocampal dentate gyrus from CON- and AIE-treated animals across exercise conditions. Scale bar = 50 μm. Data are presented as mean ± SEM. ^**^p < 0.01.

Increased progenitor cell death is an important mechanism of ethanol inhibition of hippocampal neurogenesis (Crews and Nixon, 2009). Assessment of cleaved caspase 3, a marker of cell death, was characterized by darkly stained cell bodies throughout the hippocampal subgranular zone. A 2 × 2 ANOVA (Treatment × Exercise) revealed that wheel running significantly decreased cleaved caspase 3 expression in the adult hippocampal dentate gyrus [main effect of Exercise: *F*_(1, 28)_ = 46.9, *p* < 0.01]. *Post-hoc* analysis of the significant interaction of Treatment × Exercise [*F*_(1, 28)_ = 13.4, *p* < 0.01] revealed that cleaved caspase 3+IR cells were increased by 34% (±5%) in the AIE-treated animals (Tukey's HSD: *p* < 0.01). Further, wheel running alone tended to reduce cleaved caspase 3+IR, but clearly blocked the AIE-induced increase (Tukey's HSD: *p* < 0.01; see Figure 5). Thus, exercise prevented the AIE-induced increase of cleaved caspase 3+IR that likely contributes to the prevention of inhibition of hippocampal neurogenesis.

### Voluntary wheel running prevents the AIE-induced increase of pNF-κB p65+IR and induction of neuroimmune genes in adulthood

**Figure 6:**
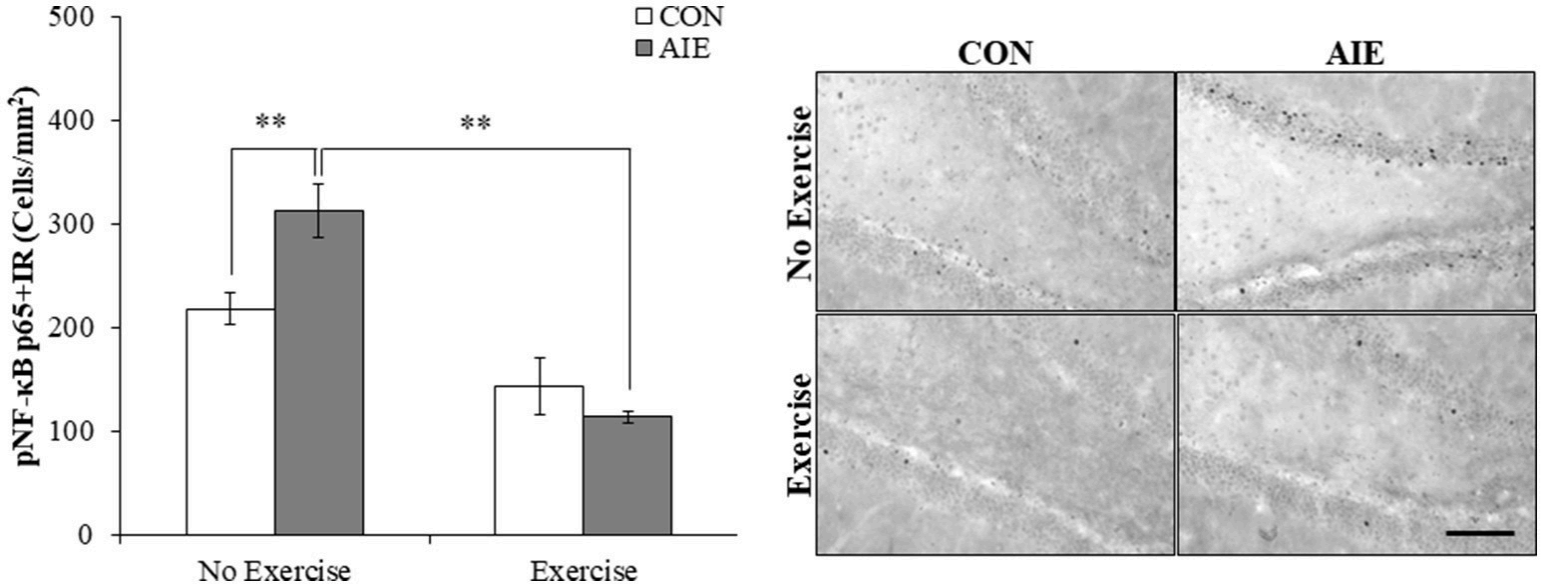
Voluntary wheel running prevented the adolescent intermittent ethanol (AIE)-induced phosphorylation of the proinflammatory transcription factor nuclear factor kappa-light-chain-enhancer of activated B cells p65 (phosphorylated [pNF-κB p65]) in the adult hippocampal dentate gyrus. Modified unbiased stereological quantification of pNF-κB p65+IR cells revealed a 40% (±12%) increase in the adult (P80) hippocampal dentate gyrus of AIE-treated animals, relative to CONs. Interestingly, subjects in the AIE treatment group that were exposed to voluntary wheel running from P24 to P80 did not evidence the observed increase of pNF-κB p65+IR cells, relative to AIE animals. Representative photomicrographs of pNF-κB p65+IR cells in the adult hippocampal dentate gyrus from CON- and AIE-treated animals across exercise conditions. Scale bar = 100 μm. Data are presented as mean ± SEM. ^**^p < 0.01.

We next investigated the impact of wheel running exercise on the AIE-induced increase of pNF-κB p65+IR, a marker of transcription activation, and NF-κB proinflammatory target gene mRNA. Analysis of the pNF-κB p65 data with a 2 × 2 ANOVA (Treatment × Drug) revealed a significant interaction of Treatment × Exercise [*F*_(1, 27)_ = 8.6, *p* < 0.01] wherein pNF-κB p65+IR was increased by 40% (±12%) in the AIE-treated animals (Tukey's HSD: *p* < 0.01), relative to CONs. Further, wheel running alone reduced pNF-κB p65+IR (Tukey's HSD: *p* < 0.01) in the CONs and completely prevented the AIE-induced increase of pNF-κB p65+IR (Tukey's HSD: *p* < 0.01; see Figure 6). Interestingly, expression of pNF-κB p65+IR across groups was negatively correlated with doublecortin+IR (*r* = −0.61, *N* = 28, *p* < 0.01; see Supplementary Figure 2A) and nestin+IR (*r* = −0.45, *N* = 29, *p* < 0.05; see Supplementary Figure 2B) consistent with a neuroimmune mechanism contributing to the AIE-induced loss of neural progenitors and neurogenesis. Further, pNF-κB p65+IR was positively correlated with expression of cleaved caspase 3 (*r* = 0.69, *N* = 29, *p* < 0.01; see Supplementary Figure 2C) suggesting that an immune mechanism is associated with the increased cell death observed in the hippocampus that is prevented by voluntary exercise. Expression of neuroimmune mRNAs in the adult brain were assessed using individual 2 × 2 ANOVAs (Treatment × Exercise) (see Table 3). Analysis of the tumor necrosis factor α (TNFα) mRNA data revealed a significant interaction [*F*_(1, 28)_ = 27.6, *p* < 0.01] such that AIE treatment led to a long-term 29% (±4%) increase of TNFα mRNA in the adult brain, relative to CONs (Tukey's HSD: *p* < 0.01) while wheel running prevented the AIE-induced increase of TNFα (Tukey's HSD: *p* < 0.01). Assessment of Toll-like receptor 4 (TLR4) mRNA revealed a significant interaction [*F*_(1, 27)_ = 11.6, *p* < 0.01] wherein AIE treatment led to a long-term 25% (±7%) increase of TLR4 mRNA in the adult brain, relative to CONs (Tukey's HSD: *p* ≤ 0.05). Assessment of Toll-like receptor 4 (TLR4) mRNA revealed that the AIE-induced increase in TLR4 mRNA is prevented by wheel running (Tukey's HSD: *p* < 0.01; see Table 3). While assessment of monocyte chemoattractant protein-1 (MCP-1) mRNA revealed a significant interaction [*F*_(1, 28)_ = 5.2, *p* < 0.05], *post-hoc* analysis did not reveal an effect of AIE or exercise on MCP-1 mRNA. Analysis of nuclear factor of kappa light polypeptide gene enhancer in B-cells inhibitor, alpha (IκBα) mRNA revealed a significant interaction [*F*_(1, 28)_ = 14.1, *p* < 0.01] such that AIE treatment led to a long-term 60% (±18%) increase of IκBα mRNA in the adult brain, relative to CONs (Tukey's HSD: *p* < 0.01) and wheel running prevented the AIE-induced increase of IκBα (Tukey's HSD: *p* < 0.05). Voluntary exercise had little effect alone on the genes assessed in the periphery with the exception of HMGB1, which is not a NF-κB target gene and was increased by voluntary exercise in the blood, brain, and liver (see Supplementary Figure 1). Thus, wheel running prevented the AIE-induced increase of pNF-κB p65 in the adult hippocampal dentate gyrus, which might contribute to the beneficial effects of exercise on neurogenesis.

**Table 3:** Effect of adolescent intermittent ethanol (AIE) and/or exercise on expression of neuroimmune signaling genes in the adult brain (P80).

**Gene**	**CON/No exercise**	**AIE/No exercise**	**CON/exercise**	**AIE/exercise**
*HMGB1*	100 ± 8	167 ± 6^**^	154 ± 9^**^	131 ± 10^*^^#^
*TNFα*	100 ± 7	129 ± 4^**^	116 ± 5	94 ± 8^##^
*MCP-1*	100 ± 10	134 ± 11	111 ± 12	95 ± 11
*TLR4*	100 ± 5	125 ± 7^*^	100 ± 7	81 ± 6^##^
*IL-4*	100 ± 16	116 ± 16	136 ± 22	62 ± 4
*IL-10*	100 ± 13	423 ± 217	102 ± 13	74 ± 12
*TGFβ*	100 ± 4	128 ± 7	67 ± 2	81 ± 2
*IκBα*	100 ± 3	160 ± 18^**^	108 ± 6	100 ± 7^##^

*Data are presented as % of CON/No Exercise*;

* *p < 0.05*;

** *p < 0.01, relative to CON/No Exercise*;

# *p < 0.05*;

## *p < 0.01, relative to AIE/No Exercise*.

### Treatment with the anti-inflammatory drug indomethacin prevents the AIE-induced loss of neurogenesis, increased cell death, and increased phosphorylated NF-κB p65 in the hippocampal dentate gyrus

**Figure 7:**
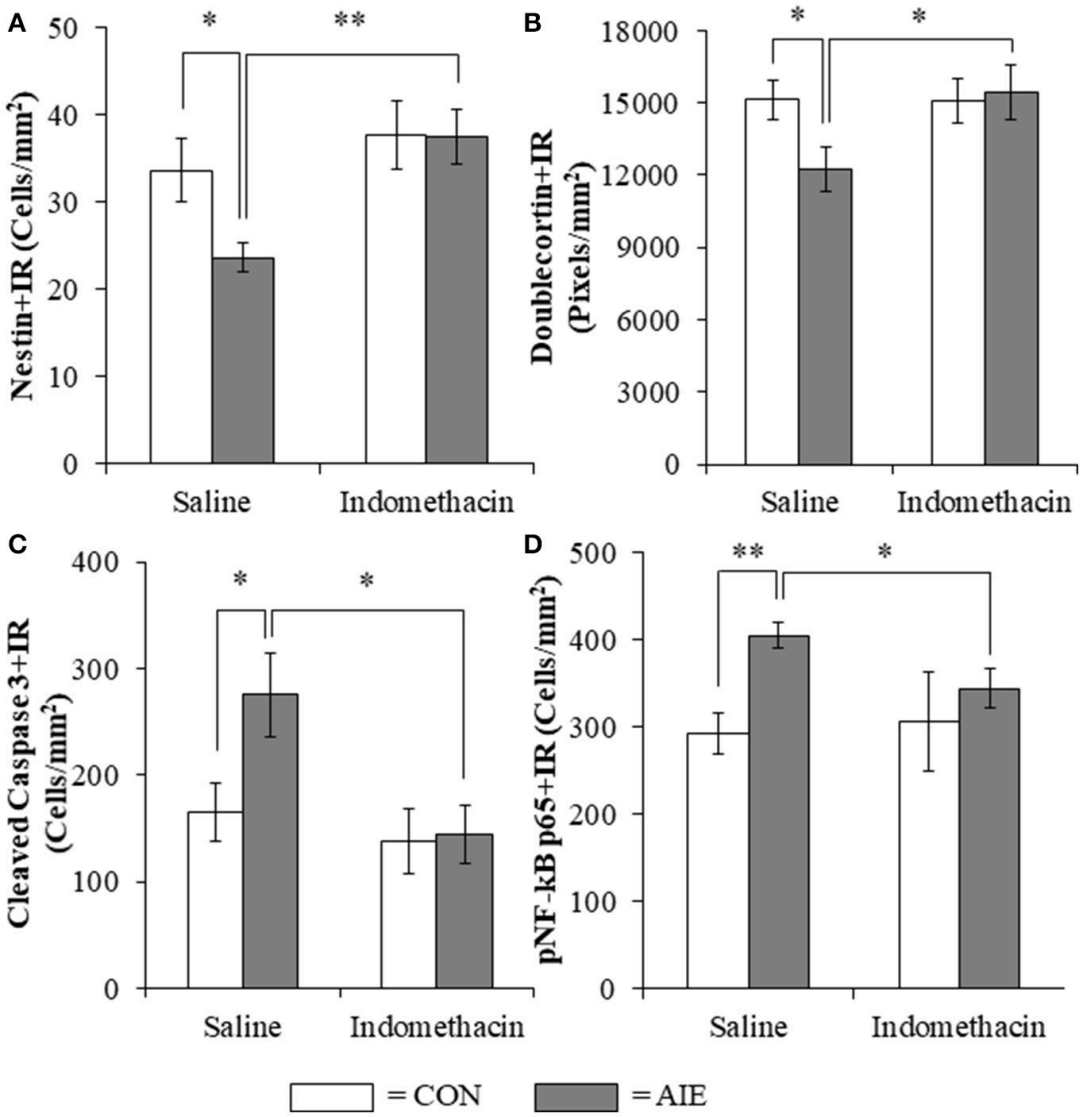
Indomethacin treatment prevented the adolescent intermittent ethanol (AIE)-induced loss of neurogenesis markers, the increase of the cell death marker cleaved caspase 3, and phosphorylation of the proinflammatory transcription factor nuclear factor kappa-light-chain-enhancer of activated B cells p65 (phosphorylated [pNF-κB p65]) in the hippocampal dentate gyrus. **(A)** Modified unbiased stereological assessment revealed a 30% (±5%) reduction of the neural progenitor marker nestin in the hippocampal dentate gyrus of late adolescent (P56) AIE-treated animals, relative to CONs. Treatment with indomethacin prevented the AIE-induced loss of nestin+ cells. **(B)** Quantification of doublecortin pixel density revealed a 21% (±6%) reduction in the hippocampal dentate gyrus of AIE-treated animals, relative to CONs. Subjects in the AIE treatment group that received indomethacin treatment did not evidence the AIE-induced loss of doublecortin+IR, relative to AIE animals. **(C)** Modified unbiased stereological quantification revealed a 66% (±24%) increase of the cell death marker cleaved caspase 3 in the hippocampal dentate gyrus of AIE-treated animals, relative to CONs. Subjects in the AIE treatment group that received indomethacin treatment did not evidence the observed increase of cleaved caspase 3+IR cells, relative to AIE animals. **(D)** Modified unbiased stereological quantification of pNF-κB p65+IR cells revealed a 38% (±5%) increase in the hippocampal dentate gyrus of AIE-treated animals, relative to CONs. Furthermore, subjects in the AIE treatment group that received indomethacin during AIE did not evidence the increase of pNF-κB p65+IR, relative to AIE animals. Data are presented as mean ± SEM. ^*^p < 0.05, ^**^p < 0.01.

Previous studies have found that the anti-inflammatory drug indomethacin (Sung et al., 2004) can block adolescent ethanol-induced inflammatory gene induction, neurodegeneration, and behavioral deficits (Pascual et al., 2007). Thus, we administered indomethacin during AIE (4.0 mg/kg, i.p., 30 min prior to each AIE treatment) and tested the hypothesis that blocking neuroimmune signals during AIE would prevent the loss of hippocampal neurogenesis. We observed an 18% (±6%) reduction of Ki-67+IR cells in the AIE-treated animals (Tukey's HSD: *p* < 0.05) and found that indomethacin did not prevent the loss of Ki-67+IR cells (data not shown). Assessment of the nestin data with a 2 × 2 (Treatment × Drug [Saline vs. Indomethacin]) ANOVA revealed that indomethacin increased expression nestin+IR [main effect of Drug: *F*_(1, 24)_ = 7.8, *p* = 0.01]. While we did not observe a significant interaction, follow-up analyses revealed a 30% (±5%) reduction of nestin+IR cells in the AIE-treated animals relative to CONs [one-way ANOVA: *F*_(1, 12)_ = 6.1, *p* < 0.05], no effect of indomethacin alone, and indomethacin prevention of the AIE-induced loss of nestin+IR cells [*F*_(1, 12)_ = 15.0, *p* < 0.01; see Figure 7A]. Analysis of the doublecortin data with a 2 × 2 ANOVA did not yield a significant interaction, but follow-up analyses revealed that AIE caused a 21% (±6%) reduction of doublecortin+IR in the hippocampal dentate gyrus relative to CONs [one-way ANOVA: *F*_(1, 12)_ = 5.5, *p* < 0.05], no effect of indomethacin alone, and indomethacin prevention of the AIE-induced loss of doublecortin+IR [one-way ANOVA: *F*_(1, 12)_ = 4.6, *p* ≤ 0.05; see Figure 7B]. Assessment of the cleaved caspase 3 data with a 2 × 2 ANOVA revealed that indomethacin reduced expression of cleaved caspase 3+IR [main effect of Drug: *F*_(1, 24)_ = 6.3, *p* < 0.05]. While we did not observed a significant interaction, follow-up analyses revealed that AIE led to a significant 66% (±24%) increase of cleaved caspase 3+IR in the AIE animals relative to CONs [one-way ANOVA: *F*_(1, 12)_ = 5.2, *p* < 0.05], no effect of indomethacin alone, and indomethacin prevention of the AIE-induced increase of cleaved caspase 3+IR [one-way ANOVA: *F*_(1, 12)_ = 7.4, *p* < 0.05; see Figure 7C]. Interestingly, cleaved caspase 3+IR across groups was negatively correlated with expression of doublecortin (*r* = −0.48, *N* = 28, *p* = 0.01) consistent with increased cell death contributing to the reduction of doublecortin+IR. Assessment of the pNF-κB p65 data with a 2 × 2 ANOVA revealed that AIE treatment increased expression of pNF-κB p65+IR [main effect of Treatment: *F*_(1, 23)_ = 5.0, *p* < 0.05]. While we did not observed a significant interaction, follow-up analyses revealed that AIE led to a significant 38% (±5%) increase of pNF-κB p65+IR in the AIE animals relative to CONs [one-way ANOVA: *F*_(1, 12)_ = 5.2, *p* < 0.05], no effect of indomethacin alone, and indomethacin prevention of the AIE-induced increase of pNF-κB p65+IR [one-way ANOVA: *F*_(1, 12)_ = 7.4, *p* < 0.05; see Figure 7D]. Interestingly, we found that pNF-κB p65+IR across groups was negatively correlated with expression of doublecortin (*r* = −0.42, *N* = 27, *p* < 0.05) and nestin (*r* = −0.43, *N* = 27, *p* < 0.05) suggesting that increased expression of pNF-κB p65 might contribute to the AIE-induced reduction of hippocampal neurogenesis. Taken together, the anti-inflammatory drug indomethacin prevented the AIE-induced loss of neurogenesis and concomitant increase of cleaved caspase 3 and pNF-κB p65 supporting the hypothesis that neuroimmune inflammatory signaling induced by AIE contributes to the loss of adult neurogenesis.

### Lipopolysaccharide treatment mimicked the AIE-induced loss of neurogenesis, increased cell death, and increased phosphorylated NF-κB p65 in the adult hippocampus

**Figure 8:**
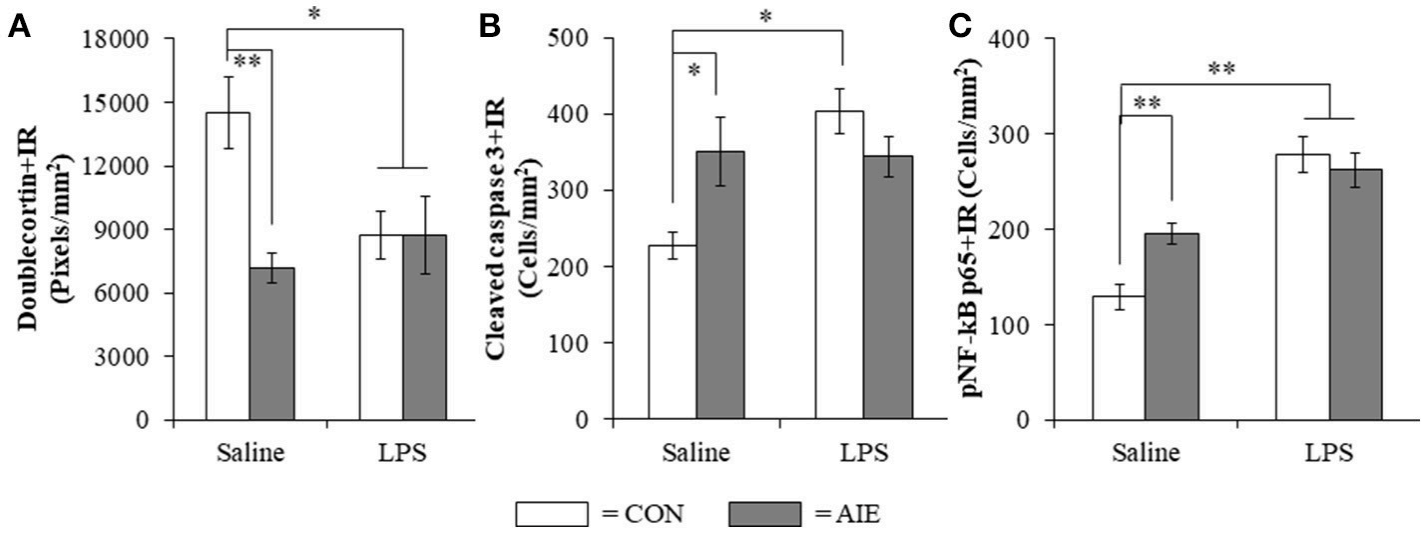
Lipopolysaccharide (LPS) treatment mimicked the AIE-induced loss of doublecortin, and increased expression of cleaved caspase 3 and phosphorylation of the proinflammatory transcription factor nuclear factor kappa-light-chain-enhancer of activated B cells p65 (phosphorylated [pNF-κB p65]) in the adult hippocampal dentate gyrus. **(A)** Quantification of doublecortin pixel density in the hippocampal dentate gyrus of adult rats (P80) revealed a significant reduction in AIE- (49% [±5%]), CON+LPS- (41% [±8%]), and AIE+LPS-treated animals (41% [±12%]), relative to CONs. **(B)** Modified unbiased stereological quantification of cleaved caspase 3+IR cells in the hippocampal dentate gyrus of adult rats revealed a significant increase in AIE- (54% [±20%]), CON+LPS-treated animals (77% [±13%]), and a tread toward an increase in AIE+LPS-treated animals (51% [±11%]), relative to CONs. **(C)** Modified unbiased stereological quantification of pNF-κB p65+IR cells in the hippocampal dentate gyrus of adult rats revealed a significant increase in AIE- (52% [±14%]), CON+LPS- (116% [±9%]), and AIE+LPS-treated animals (104% [±14%]), relative to CONs. Data are presented as mean ± SEM. ^*^p < 0.05, ^**^p < 0.01.

To determine if neuroimmune activation recapitulates AIE-induced hippocampal neuropathology, a separate cohort of CON- and AIE-treated rats received a single dose of LPS (1.0 mg/kg i.p.) and were sacrificed 10 days later on P80. Interestingly, LPS treatment mimicked some, but not all AIE effects on neurogenesis. Consistent with our previous experiments, AIE treatment reduced Ki-67+IR (35% [±5%]; one-way ANOVA: *F*_(1, 12)_ = 9.5, *p* = 0.01) and nestin+IR (45% [±9%]; one-way ANOVA: *F*_(1, 12)_ = 7.8, *p* < 0.05) cells in the adult hippocampus whereas LPS did not alter expression of neural proliferation markers (i.e., Ki-67 and nestin; data not shown). In contrast, assessment of doublecortin using a 2 × 2 (Treatment × Drug [Saline vs. LPS]) revealed a significant interaction [*F*_(1, 128)_ = 6.1, *p* < 0.05] such that doublecortin+IR was reduced by 49% (±5%) in the AIE subjects (Tukey's HSD: *p* < 0.01), 41% (±8%) in the LPS alone subjects (Tukey's HSD: *p* < 0.05), and 41% (±12%) in the AIE+LPS subjects (Tukey's HSD: *p* < 0.05), relative to CONs (see Figure 8A). Similarly, *post-hoc* analysis of the significant interaction of cleaved caspase 3 [*F*_(1, 26)_ = 8.7, *p* < 0.01] revealed that cleaved caspase 3+IR was increased by 54% (±20%) in the AIE subjects (Tukey's HSD: *p* ≤ 0.05), 77% (±13%) in the LPS alone subjects (Tukey's HSD: *p* < 0.01), and trended toward an increase in the AIE+LPS subjects (51% [±11%]; Tukey's HSD: *p* = 0.06), relative to CONs (see Figure 8B). Cleaved caspase 3+IR across groups was negatively correlated with expression of doublecortin (*r* = −0.37, *N* = 30, *p* < 0.05) suggesting that increased cell death is associated with the AIE-induced loss of neurogenesis. As expected, *post-hoc* analysis of the significant interaction of pNF-κB p65 [*F*_(1, 28)_ = 7.0, *p* < 0.05] revealed that pNF-κB p65+IR was increased by 51% (±14%) in the AIE-treated animals, relative to CONs (Tukey's HSD: *p* < 0.05). Lipopolysaccharide treatment resulted in a 116% (±9%) and 104% (±14%) increase of pNF-κB p65+IR cells in CON- (Tukey's HSD: *p* < 0.01) and AIE-treated (Tukey's HSD: *p* < 0.01) animals, respectively, relative to CONs (see Figure 8C). Interestingly, expression of pNF-κB p65 across groups was positively correlated with cleaved caspase 3+IR (*r* = 0.46, *N* = 30, *p* = 0.01) consistent with a neuroimmune mechanism increasing adult hippocampal dentate gyrus cell death. Taken together, these data support our hypothesis that the inflammagen LPS mimics AIE induction of hippocampal neuroimmune signaling, increased cell death, and loss of adult neurogenesis.

## Discussion

**Figure 9:**
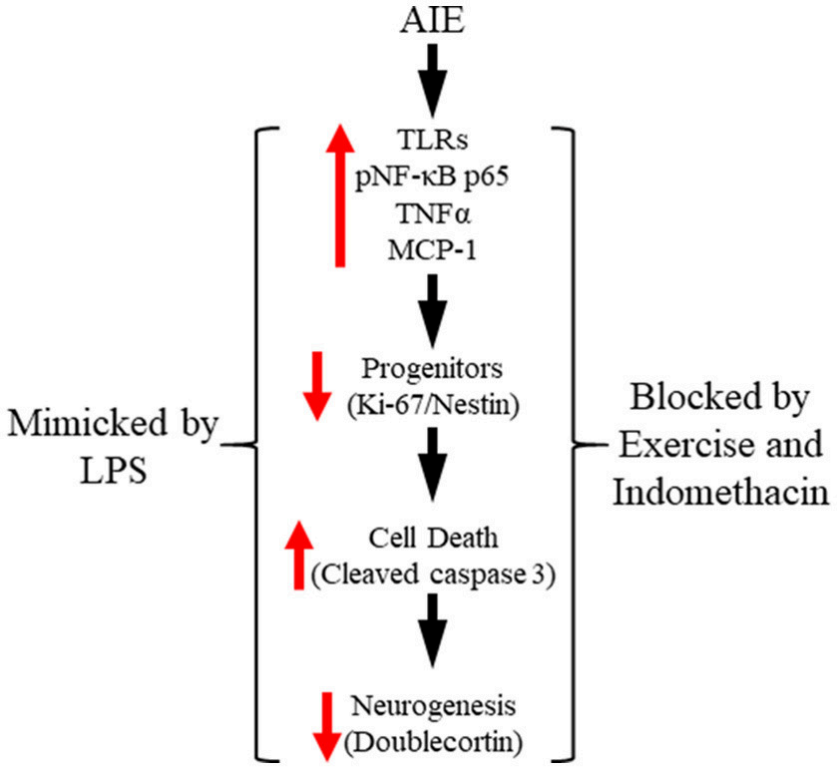
Proposed mechanism of adolescent intermittent ethanol (AIE)-induced loss of hippocampal neurogenesis. Adolescent binge ethanol exposure upregulations Toll-like receptor (TLR) expression in the hippocampus resulting in increased phosphorylation of the proinflammatory transcription factor nuclear factor kappa-light-chain-enhancer of activated B cells p65 (phosphorylated [pNF-κB p65]). This in turn results in the induction of neuroimmune signaling molecules, including tumor necrosis factor alpha (TNFα) that activate positive loops of amplification that persist into adulthood (Crews and Vetreno, 2016). The persistent proinflammatory neuroimmune induction results in diminished neurogenesis and increased cell death that persists into adulthood. Importantly, exposure to either voluntary wheel running, which conveys immune-modulatory effects, or treatment with the anti-inflammatory drug indomethacin restores hippocampal neurogenesis by preventing the AIE-induced increase of cell death (i.e., cleaved caspase 3) as well as increased phosphorylation of pNF-κB p65. Moreover, treatment with the endotoxin lipopolysaccharide (LPS) mimicked the AIE-induced loss of neurogenesis while increasing expression of cleaved caspase 3 and pNF-κB p65. Together, these data suggest that increased expression of neuroimmune molecules increases cell death in the hippocampus resulting in the persistent AIE-induced loss of neurogenesis.

In the present study, we extend our previous findings on AIE inhibition of adult neurogenesis with data supporting neuroimmune involvement in the persistent loss of hippocampal neurogenesis. We report here for the first time that AIE treatment, which models human adolescent binge drinking, causes long-term increases of multiple proinflammatory TLR mRNAs in the adult (i.e., P80) hippocampus. Further, using pNF-κB p65 as an indicator of activation of the nuclear transcription factor target of TLR activation, we report here for the first time increased expression of pNF-κB p65+IR in the post-mortem human alcoholic hippocampal dentate gyrus that negatively correlates with age of drinking onset and in AIE-treated rats correlated with increased expression of the cell death marker cleaved caspase 3. Toll-like receptors and NF-κB are expressed in multiple cell types (Crews et al., 2017; Lawrimore and Crews, 2017). We report here that pNF-κB p65+IR colocalized with both immature doublecortin+IR neurons and Iba-1+IR microglia, with AIE increasing the percentage of Iba-1+IR microglia expressing pNF-κB p65 consistent with microglial sensitization and increased proinflammatory signaling following AIE. We report here for the first time that exposure to voluntary wheel running from P24 to P80 prevents the AIE-induced loss of neurogenic markers (i.e., nestin and doublecortin), increased expression of the cell death marker cleaved caspase 3 and pNF-κB p65, and induction of neuroimmune NF-κB target genes, including *TNF*α and *I*κ*B*α. These studies add to previously published studies finding that exercise blunts neuroimmune signaling responses to ischemic pathology (Ma et al., 2013). We also add to previous studies reporting that the anti-inflammatory drug indomethacin blocks adolescent ethanol-induced persistent increase of neuronal death (Pascual et al., 2007) our finding of indomethacin prevention of the AIE-induced loss of neurogenesis. Further, we report that LPS treatment of rats mimics AIE-induced persistent increases in pNF-κB p65 and the cell death marker cleaved caspase 3 as well as the loss of markers of neurogenesis. Taken together, these data support the hypothesis that adolescent binge ethanol-induced upregulation of neuroimmune signaling molecules contributes to the persistent loss of neurogenesis in the adult hippocampus (see Figure 9).

Toll-like receptors are a class of pattern recognition receptor that respond to exogenous and endogenous signaling molecules (Bianchi, 2007) by mounting a proinflammatory neuroimmune response. In the present study, we found that AIE led to the long-term upregulation of TLRs 1–8 (with the exception of TLR3) in the adult hippocampus. This study replicates and extends our previous discoveries that AIE increases TLR4 mRNA in the adult hippocampus (Vetreno and Crews, 2015), TLR3 and TLR4 in the adult prefrontal cortex (Vetreno and Crews, 2012), and multiple TLR mRNAs (i.e., TLR1-TLR8) in the adult cerebellum (Crews et al., 2017). Activation of TLR cascades lead to activation of the nuclear transcription factor NF-κB (Kawai and Akira, 2007). Our findings of increased expression of pNF-κB p65 in the hippocampal dentate gyrus of adult AIE-treated animals and human alcoholics, the latter of which was associated with an earlier adolescent age of drinking onset, is consistent with adolescent alcohol exposure resulting in long-term upregulation of neuroimmune signaling in the hippocampus. Our findings are consistent with previous studies reporting acute AIE-induced (i.e., 24 h post-AIE) upregulation of pNF-κB p65 in the mouse cortex that persists into young adulthood (i.e., P64) (Montesinos et al., 2016) and upregulation of NF-κB target genes in the post-mortem human alcoholic prefrontal cortex (Okvist et al., 2007). While NF-κB signaling cascades have largely been ascribed to glial cells with a particular emphasis on microglia, NF-κB p65 is also constitutively expressed in neurons of the hippocampus and cortex (Kaltschmidt et al., 1994). In the present study, we found that pNF-κB p65 was highly co-expressed in immature doublecortin neurons of the hippocampal dentate gyrus, which is consistent with a role of NF-κB in plasticity and neurogenesis (for review, see Bortolotto et al., 2014). Interestingly, we observed a higher incidence of pNF-κB p65 colocalization in microglial cells of AIE-treated animals. These findings are consistent with AIE and human alcoholism causing microglial activation (He and Crews, 2008; McClain et al., 2011; Rubio-Araiz et al., 2016). However, our studies of ethanol induction of multiple proinflammatory NF-κB target genes, including *TNF*α, *IL-1*β, and *MCP-1* suggest that multiple brain cell types are involved in ethanol induction of neuroimmune signaling (Lawrimore and Crews, 2017; Walter and Crews, 2017). Interestingly, inhibition of microglial activation with minocycline dose-dependently blocks ethanol-induced proinflammatory cytokine induction (e.g., TNFα and IL-1β; Zou and Crews, 2014) and recovers immunostimulant-induced loss of neurogenesis (Mattei et al., 2014). Taken together, our observations that AIE causes a long-term upregulation of TLRs and pNF-κB p65 following cessation of ethanol exposure suggests that adolescent ethanol exposure leads to neuroimmune activation that persist into adulthood (Crews and Vetreno, 2011) and contributes to the AIE-induced loss of neurogenesis in the adult hippocampus.

Hippocampal neurogenesis is a dynamic process that is elevated in adolescence and declines with age into senescence, and is highly susceptible to modulation of environmental influences. Previous studies have found that voluntary exercise increases neurogenesis in senescent mice (van Praag et al., 2005; Gibbons et al., 2014) and following conditioned running in senescent F344 rats (Speisman et al., 2013). Although we have previously reported that AIE inhibition of neurogenesis persists relative to the age-related decline in control animals from youth to mature adulthood (i.e., P56–P220) (Vetreno and Crews, 2015), we did not find that voluntary exercise increased expression of neurogenesis markers (i.e., Ki-65, nestin, or doublecortin) or reduced cleaved caspase 3+IR in young adult CONs. Although we have previously reported that AIE inhibition of neurogenesis persists relative to the age-related decline in control animals from youth to mature adulthood (i.e., P56–P220) (Vetreno and Crews, 2015), we did not find that voluntary exercise increased expression of neurogenesis markers (i.e., Ki-65 or doublecortin) in young adult CONs. Previous studies from our laboratory in adult mice found that exercise increased levels of neurogenesis in controls and prevented ethanol inhibition of neurogenesis (Crews et al., 2004). Other studies in mice have found that exercise can increase neurogenesis in controls (Nishijima et al., 2017; Sack et al., 2017). These data contrast work from Klein et al. (2016) that exercise did not increase expression of doublecortin in exercising control mice and that exercise did not increase Ki-67 in adult rats (Leasure and Nixon, 2010). To our knowledge, our study is the first to assess neurogenesis following adolescent exercise exposure in rats and we found that exercise reduced cleaved caspase 2, a marker of cell death, and restored the AIE-induced loss of doublecortin, Ki-67, and nestin to control levels. Consistent with our findings of an immunomodulatory effect of exercise, other studies have reported that exercise creates resilience against insults by blunting neuroimmune responses. For example, ischemia induced by middle cerebral artery occlusion in rats increases TLR, NF-κB, and proinflammatory cytokine gene expression that is blunted by treadmill exercise (Ang et al., 2004; Ma et al., 2013). This study extends previous findings to show that wheel running blunts the AIE-induced loss of the cell proliferation marker Ki-67, and prevents the loss of the progenitor cell marker nestin and the immature neuron marker doublecortin in the adult hippocampus. Consistent with the present data, wheel running restores neurogenic markers in adult mice exposed to an acute ethanol self-administration paradigm in adulthood (Crews et al., 2004). In addition, we found that voluntary exercise prevented the AIE-induced increase of the cell death marker cleaved caspase 3 while blocking the increase of pNF-κB p65 as well as induction of NF-κB target genes, including *TNF*α and *I*κ*B*α in the adult brain. Consistent with a neuroimmune mechanism underlying the AIE-induced loss of neurogenesis was our observation that pNF-κB p65+IR was negatively correlated with expression of neurogenic markers (i.e., nestin and doublecortin) and positively correlated with expression of cleaved caspase 3. Of particular import, exercise prevented the AIE induction of IκBα, which is induced by NF-κB p65 activation in an autoregulatory fashion leading to restoration of intracellular IκBα binding to the p65 subunit and inhibition of NF-κB activity (Sun et al., 1993), indicative of long-term activation of the proinflammatory NF-κB p65 signaling pathway in the AIE-treated adult brain. Further, application of recombinant IL-6 or TNFα to cultured neuroprogenitor precursor cells reduced neurogenesis marker expression (i.e., doublecortin Monje et al., 2003) through a process involving increased cell death (Monje et al., 2003; Guadagno et al., 2013). These data support involvement of the neuroimmune system in the AIE-induced loss of hippocampal neurogenesis through a process involving increased cell death. In contrast to our findings in the CNS, we did not observe an AIE-induced long-term upregulation of immune genes in the periphery (i.e., liver, spleen, or serum), which is consistent with findings from our laboratory of persistent neuroimmune induction in brain, but not serum or liver, following chronic ethanol exposure or a single dose of endotoxin in adult mice (Qin et al., 2007, 2008). Taken together, these data reveal that voluntary exercise prevents the AIE-induced loss of neurogenesis and supports the hypothesis of a neuroimmune mechanism underlying the persistent loss of hippocampal neurogenesis.

Although the data support exercise prevention of neuroimmune signaling as a mechanism of AIE-induced loss of neurogenesis, exercise confers additional beneficial effects to the CNS (e.g., increased neurotrophins, increased cerebral blood flow) that may be independent or a primary mechanism related to reversal of the neuroimmune signaling and the persistent loss of hippocampal neurogenesis. Previous studies have found that AIE reduces hippocampal brain-derived neurotrophic factor (BDNF)+IR in CA1-CA3, but not dentate gyrus, that was recovered by treatment with a HDAC inhibitor and accompanied by restoration of hippocampal neurogenesis (Sakharkar et al., 2016). Forced exercise in rats has been found to increase hippocampal NGF and the NGF p75 receptor expression (Ang et al., 2003) as well as to reduce neuroimmune gene induction following MCAO ischemia (Ang et al., 2004). In a study of adolescent rat brain irradiation which is known to increase oxidative stress and innate immune signaling, exercise blunted the loss of neurogenesis and increased expression of BDNF and TrkA, consistent with other studies findings exercise creates resilience against insults by both increasing trophic and reducing neuroimmune signaling.

While exercise is known to create resilience, the combined tropic and anti-inflammatory actions confound interpretation of mechanism. To more directly assess the neuroimmune hypothesis, we administered the non-steroidal anti-inflammatory drug indomethacin during AIE treatment. Our data extend previous studies showing that indomethacin blocks AIE-induced apoptotic DNA fragmentation and caspase 3 activity in the rat neocortex, hippocampus, and cerebellum 24 h after the last ethanol treatment in a rat model of AIE (Pascual et al., 2007). We found that indomethacin treatment blocked the AIE-induced increase of pNF-κB p65 in the hippocampal dentate gyrus and prevented the loss of neurogenesis markers (i.e., doublecortin and nestin) as well as the increase of the cell death marker cleaved caspase 3. Somewhat surprising was our finding that indomethacin did not prevent the AIE-induced loss of the cell proliferation marker Ki-67. It is possible that this is due to the anti-proliferative effects of indomethacin and its use as a treatment for various cancers of the CNS (Bernardi et al., 2009). Consistent with our data, Monje et al. (2003) found that indomethacin treatment blocked LPS-induced loss of doublecortin in the rat hippocampal dentate gyrus. In the present study, we also found that activation of the neuroimmune system using the proinflammatory TLR4 activator LPS caused a loss of doublecortin, and increased expression of cleaved caspase 3 and pNF-κB p65 in the hippocampal dentate gyrus that mimics AIE. Further, we found that expression of pNF-κB p65 was positively correlated with expression of cleaved caspase 3 suggesting that neuroimmune activation is associated with increased cell death. Previous studies have found that the anti-inflammatory drug indomethacin can block adolescent ethanol exposure-induced inflammatory gene induction, neurodegeneration, and behavioral deficits (Pascual et al., 2007). In the present study, we found that indomethacin, which can inhibit NF-κB activity (Sung et al., 2004), prevented the AIE-induced increase of pNF-κB p65, the increase of the cell death marker cleaved caspase 3, and the loss of hippocampal neurogenesis markers (i.e., doublecortin and nestin) consistent with neuroimmune activation contributing to the AIE-induced cell death and loss of neurogenesis in the adult hippocampus. Taken together, these data indicate that anti-inflammatory drug treatment blocks and neuroimmune system activation mimics the AIE-induced loss of hippocampal neurogenesis thereby supporting the hypothesis that neuroimmune signaling contributes to the AIE-induced loss of neurogenesis.

Although not the focus of the current study, hippocampal neurogenesis is critically involved in cognitive and emotive function (Madsen et al., 2003; McHugh et al., 2004; Revest et al., 2009), and the loss of neurogenesis following AIE is associated with behavioral deficits in adulthood (Ehlers et al., 2013a; Vetreno and Crews, 2015). The present data suggest that interventions aimed at restoring neurogenesis, such as exercise and indomethacin, might lead to the recovery of cognitive dysfunction in adulthood following AIE. Indeed, exercise has been shown to recover neurogenesis and cognitive deficits across various animal models of neuropathology (Speisman et al., 2013; Gibbons et al., 2014; Winocur et al., 2014). Similarly, indomethacin treatment has been reported to blunt age-related cognitive decline in middle-aged rats (McGuiness et al., 2017). Alterations in hippocampal neurogenesis are also observed in human neurodegenerative and psychological disorders (Sierra et al., 2011), which likely contribute to the cognitive and emotive dysfunction that accompany these conditions (e.g., major depressive disorder). Despite the limitations associated with measuring neurogenesis in humans, exercise training has similarly been shown to improve hippocampal-dependent memory and prevent age-related cognitive decline in humans (Hillman et al., 2008; Dery et al., 2013) consistent with the beneficial effects of exercise on neuroplasticity and hippocampal neurogenesis.

In conclusion, our data support the hypothesis that AIE-induced upregulation of neuroimmune signaling contributes to the loss of hippocampal neurogenesis. Adolescent intermittent ethanol exposure leads to long-term upregulation of proinflammatory TLRs in the hippocampus that is accompanied by increased expression of the nuclear transcription factor pNF-κB p65 and the induction several neuroimmune NF-κB target genes in the adult brain, including TNFα, MCP-1, and IκBα. Upregulation of pNF-κB p65 leads to the induction of proinflammatory cytokines and other neuroimmune signaling molecules (e.g., TNFα, MCP-1) that lead to further NF-κB activation following the cessation of ethanol exposure thereby providing support for the establishment of positive loops of neuroimmune signaling in the AIE model that persist into adulthood (Crews and Vetreno, 2011). Blockade of pNF-κB p65 expression through exposure to wheel running or administration of the anti-inflammatory drug indomethacin prevented the AIE-induced loss of neurogenesis and concomitant increased expression of the cell death marker cleaved caspase 3. Further, administration of LPS, which is a known neuroimmune activator through TLR4, mimicked the AIE-induced increase of pNF-κB p65, and loss of hippocampal neurogenesis (see Figure 9). Together, these novel findings implicate proinflammatory pNF-κB p65-neuroimmune signaling in the AIE-induced loss of hippocampal neurogenesis.

## Author contributions

RV, PR, and FC were responsible for the study concept and design. RV and CL were responsible for the data preparation and analysis. RV and FC drafted the manuscript. All authors were involved in manuscript editing and have approved the final version for publication.

### Conflict of interest statement

The authors declare that the research was conducted in the absence of any commercial or financial relationships that could be construed as a potential conflict of interest.
