# Supplementary material for: Persistent Adult Neuroimmune Activation and Loss of Hippocampal Neurogenesis Following Adolescent Ethanol Exposure: Blockade by Exercise and the Anti-inflammatory Drug Indomethacin
Source: Front Neurosci. 2018 Mar 28;12:200. doi: 10.3389/fnins.2018.00200 (PMC5882830; doi:10.3389/fnins.2018.00200)
Supplement: Supplementary file 1 [file DataShee1.docx]

**Supplemental Material**


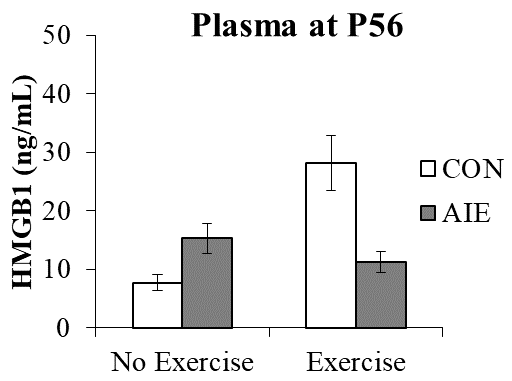

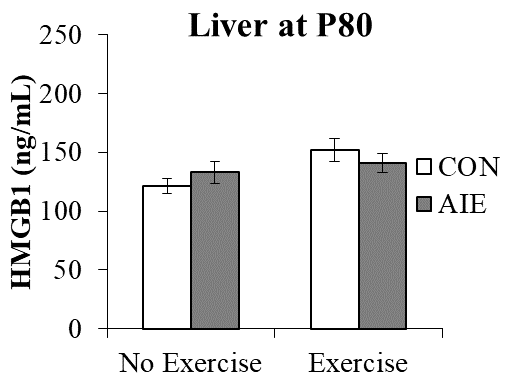


A.

B.

*

**

**Figure 1. Increased expression of high-mobility group box 1 (HMGB1) in the plasma and liver of exercising rats.** (A) Enzyme-linked immunosorbent assay (ELISA) assessment revealed increased HMGB1 plasma levels on postnatal day (P)56 by 271% (±62%) (Tukey’s HSD; *p* < 0.01) in the exercising CON animals, relative to the no exercise CONs. (B) ELISA assessment revealed that exercise exposure increased protein levels of HMGB1 in the liver, regardless of treatment condition (main effect of Exercise: *F*_(1,30)_ = 5.0, *p* < 0.05). Data are presented as mean ± SEM. * *p* < 0.05, ** *p* < 0.01.


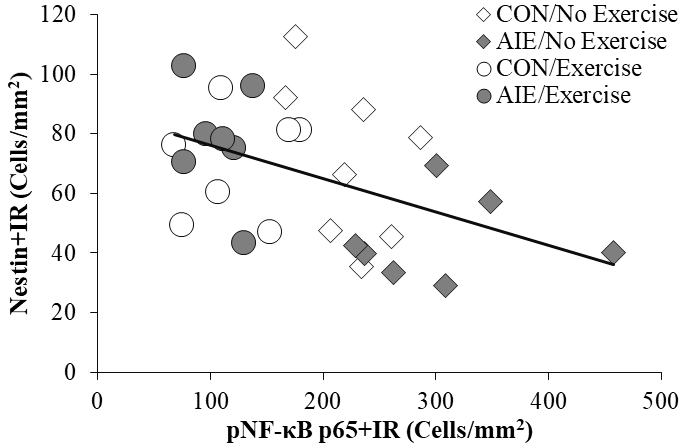


A.


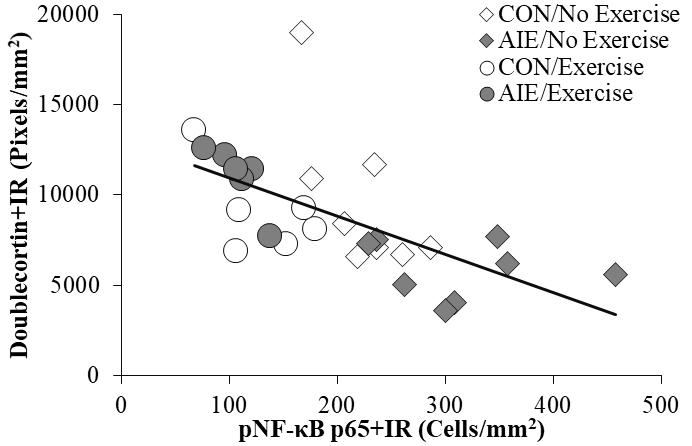


B.


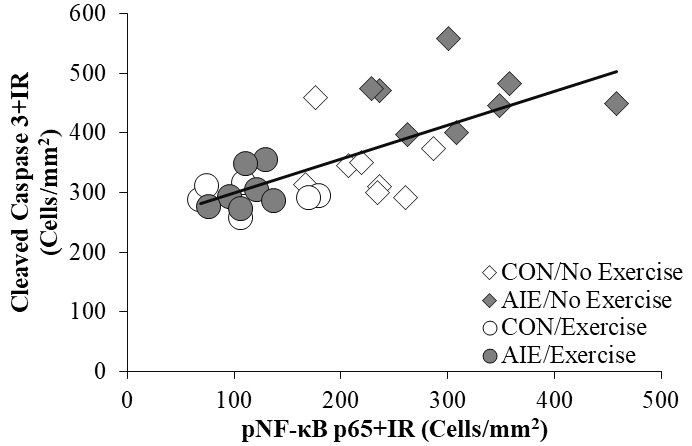


C.

**Figure 2. Expression of pNF-κB p65 is correlated with expression of nestin, doublecortin, and cleaved caspase 3 in the adult hippocampus.** (A) Expression of pNF-κB p65+IR across groups was negatively correlated with doublecortin+IR (r = -0.61, N = 28, *p* < 0.01). (B) Expression of pNF-κB p65+IR across groups was negatively correlated with nestin+IR (r = -0.45, N = 29, *p* < 0.05). (C) Expression of pNF-κB p65+IR was positively correlated with cleaved caspase 3+IR (r = 0.69, N = 29, *p* < 0.01).
